# Subcellular visualization and quantification of cyanotoxin synthesis in cyanobacteria reveals distinct compartmentation

**DOI:** 10.1038/s41598-026-47303-1

**Published:** 2026-04-19

**Authors:** Rubén Morón-Asensio, Rainer Kurmayer

**Affiliations:** 1 Research Department for Limnology, University of Innsbruck, Mondseestrasse 9, Mondsee, 5310 Austria; 2 Universität Innsbruck, Innrain 52, Innsbruck, 6020 Austria

**Keywords:** Biochemistry, Biological techniques, Biophysics, Cell biology

## Abstract

The bloom-forming cyanobacteria *Microcystis* and *Planktothrix* produce cyanotoxins such as microcystins (MCs) and anabaenopeptins (APs) through nonribosomal peptide synthesis (NRPS). Close inspection of their synthesis can aid in understanding the biological function of such cyanotoxins. MCs and APs were visualized using chemoselective labeling, and a heterogeneous distribution of fluorescence signals was observed during both time-lapse signal build-up and decline experiments. The formation of rather distinct entities was quantified using advanced imaging analysis, defining modeled entities (ME) with varying intensities but rather stable volumes, numbers and localization. The origin of MC synthesis was mostly in the cytoplasm of unicellular *Microcystis* and was confirmed through immunolabeling. In contrast, for APs in filamentous *Planktothrix*, the origin was localized in the membrane region near the septa between filament cells. Our data suggest distinct cyanotoxin biosynthesis centers in cells, differing in their localization between unicellular and multicellular cyanobacteria.

**Supplementary Information:**

The online version contains supplementary material available at 10.1038/s41598-026-47303-1.

## Main

Bloom-forming cyanobacteria, particularly the genera *Microcystis* and *Planktothrix*, are significant contributors to harmful algal blooms (cHABs) in freshwater, resulting in economic losses globally^1,2^. These genera produce cyanotoxins like microcystins (MCs) and bioactive peptides such as anabaenopeptins (APs). High cyanotoxin concentrations have been linked to growth and biomass accumulation, notably observed along Langmuir spirals^3^. While MCs inhibit eukaryotic protein phosphatases^4^, some APs inhibit proteases^5^. Despite the well-documented negative ecological and health effects of cHABs, the biological functions of cyanotoxins are not fully understood^6^, with suggested multifunctional roles for these compounds^7,8^. Both MCs and APs are synthesized by large multimodular enzymes via nonribosomal peptide synthesis (NRPS)^9,10^. Natural mutations in NRPS adenylation (A)-domains decrease amino acid activation specificity, permitting the incorporation of nonnatural amino acids (non-AAs) with azide or alkyne moieties into MC or AP molecules^10–12^ such as 4-azidophenylalanine (Phe-Az), N-propargyloxy-carbonyl-L-lysine (Prop-Lys) and O-propargyl-L-Tyrosine (Prop-Tyr). These modified molecules can be labeled with fluorophores using copper-catalyzed azide–alkyne cycloaddition (CuAAC)^13–15^ with fluorophores such as Alexa Fluor 488 (A488-azide/alkyne), carrying the chemoselective functional group, i.e., A488-alkyne for Phe-Az and A488-azide for Prop-Lys and Prop-Tyr.

An evolutionary tendency towards increased structural diversity among *Planktothrix* spp. peptide families has been observed^16–18^. The mechanism, however, differs between the individual NRPS. For MC and AP synthesis, recombination and point mutations within A-domains lead to structural diversity with chemically distinct amino acids, resulting in the co-production of multiple structural variants^10,11,16–19^. In addition, several cyanopeptolin variants were also found co-occurring in individual *Planktothrix* strains, with variation in positions adjacent to the conserved amino-hydroxy-piperidone (Ahp) which is likely synthesized by promiscuous A-domains^20,21^. Cyanopeptolin synthesis is further modified by accessory enzymes such as halogenases, methylases and sulfatases but also duplication or triplication of NRPS modules encoded by the ociA gene^22^. In contrast for aeruginosins, several accessory enzymes like halogenases, sulfotransferases, glycosyltransferases and others cause a large part of the structural variation^23^. Finally, among all the NRPS the microginin synthesis genes were found most rarely distributed and - within the genus *Planktothrix* - the only functional NRPS encoded on plasmids^22,24^. In comparison to NRPS, the two ribosomal peptide synthesis (RiPP) pathways, i.e., microviridin synthesis^25^ as well as prenylagaramide synthesis^26^ were found to be more frequently distributed. Since for RiPPs in general larger precursor peptides are produced ribosomally and subsequently modified post-translationally^25,26^ an incorporation of non-AAs analogous to promiscuous A-domains in NRPS is unlikely. In summary, the elucidation of structural variation of NRPS or RiPP synthesis among *Planktothrix* spp. allows to some extent to predict peptide modification via feeding of non-natural (clickable) amino acids^12,13^.

Recent developments include click chemistry to create a traceable cyanotoxin pool for tracking MCs/APs during time-lapse experiments^27^. This technology has been utilized to visualize toxin synthesis and to observe toxin intracellular localization. In previous studies, the labeling signals of clickable MCs in *Microcystis aeruginosa* and clickable APs in *Planktothrix agardhii* appeared as distinct entities. MCs were distributed throughout *M. aeruginosa* cells, while APs were primarily located near intercellular septa in *P. agardhii*^14,28^. This observed heterogeneous distribution might have important implications for understanding cyanotoxin synthesis regulation and their functional roles in cells.

Here, we used high-resolution microscopy and advanced imaging analysis to investigate toxin synthesis in cyanobacteria *M. aeruginosa* and *P. agardhii*. Immunofluorescence labeling demonstrated the reliability of chemoselective labeling for subcellular visualization of MCs in *M. aeruginosa*. We quantified the presence of labeling signals in distinct entities, allowing their localization at an individual scale. We show that so-called modeled entities (ME) sharply increased or decreased in signal intensity during time-lapse experiments, whereas their number per cell, volume and intracellular distribution remained relatively stable. This relative stability of ME suggests that these structures are sites of intracellular peptide synthesis rather than storage compartments for MCs or APs. The ME showed consistent intracellular distribution, differing between MCs produced in *M. aeruginosa* and APs produced in *P. agardhii*.

## Results

### Combination of click-chemistry labeling with immunolabeling of microcystins

**Fig. 1:**
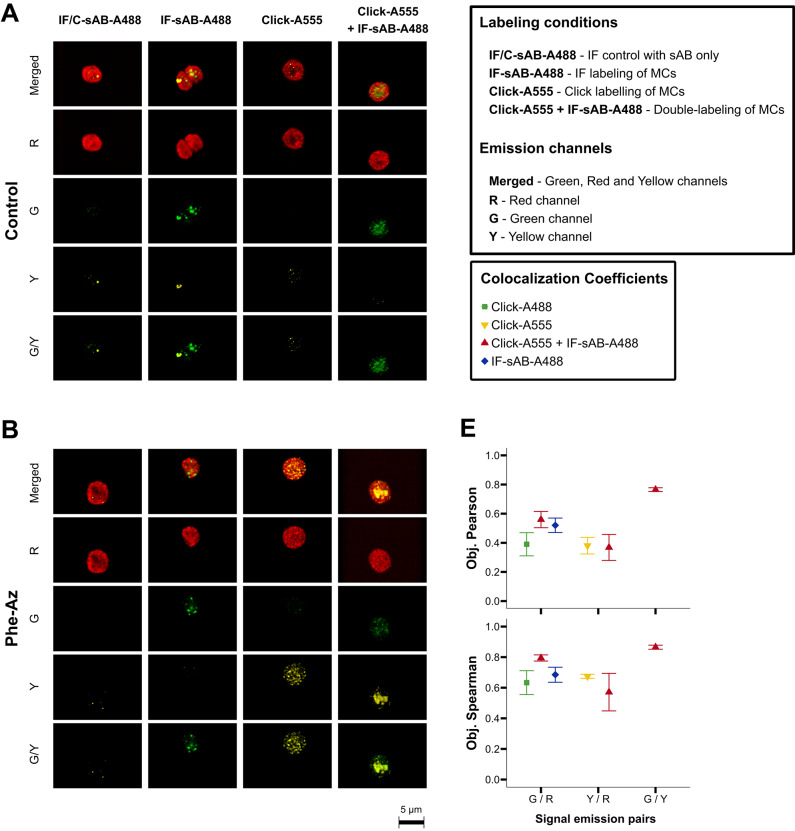

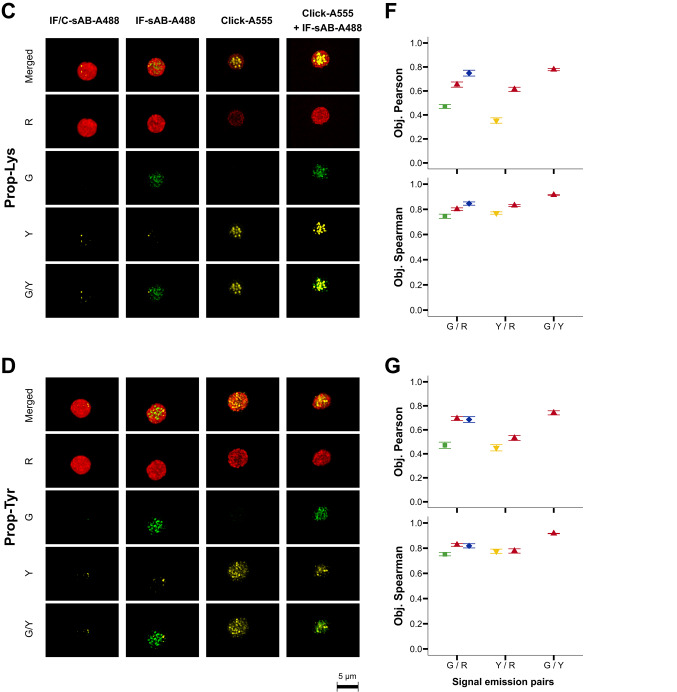
Combining immunofluorescence (IF) labeling of MCs with chemoselective visualization of MCs in cells of *M. aeruginosa* strain Hofbauer (**A–D**). For this purpose, a double-labeling protocol for chemoselective labeling of clickable MCs (yellow fluorophore, click-A555-azide/alkyne) and the subsequent immunolabeling of MCs (green fluorophore, IF-sAB-A488) was established. *M. aeruginosa* cells were grown in the presence of non-AA, either Phe-Az, or Prop-Lys, or Prop-Tyr, to enable a click-chemistry reaction between clickable MC and A555-azide/alkyne. Control cells were grown in the absence of non-AA but treated identically. For immunofluorescence a monoclonal primary antibody (binding to the Adda side chain of the MC molecule) coupled to sAB-488 was used. The specificity of the secondary antibody sAB-488 was tested in absence of primary antibody (IF/C). Natural autofluorescence (AF) is observed in red. (**E–G**) The mean ± SE colocalization coefficients (Object Pearson and Object Spearman) were calculated to quantify the spatial correlation between different emission pairs: green channel (G) vs. red channel (R), i.e., IF-sAB-A488 vs. AF; yellow channel (Y) vs. R, i.e., click-A555 vs. AF; G/Y, i.e., IF-sAB-A488 vs. click-A555. For quantitative comparison of signal intensity between G and Y for both MC and another protein (RbcL, Rubisco large subunit) see Extended Data Fig. 1.

Since a monoclonal primary antibody (binding to the Adda side chain of the MC molecule) has been used to trace MCs in cells^29^, a double-labeling protocol for chemoselective labeling of clickable MCs with Alexa Fluor 555 azide or alkyne (A555-azide/alkyne; yellow) and subsequent immunolabeling of MC or Rubisco with Alexa Fluor 488 secondary antibody (sAB-A488; green) in the same cells was established. The combination of A555-azide/alkyne and sAB-A488 enabled accurate differential labeling of MCs, maintaining low interference. Results confirmed previously reported heterogeneous structures of MCs, showing low-to-average colocalization between autofluorescence (AF) and labeled MCs^13^. Notably, significant increases in colocalization coefficients were observed between clickable MCs (A555-azide/alkyne) and immunolabeled MCs (sAB-A488) when using one of the three non-AAs to generate clickable MCs, i.e., Phe-Az, Prop-Lys, Prop-Tyr (Fig. 1), highlighting the effectiveness of click chemistry in visualizing and quantifying intracellular MC molecules.

### Signal intensity resulting from clickable cyanotoxins in cyanobacteria cells

It has been shown earlier that although *M. aeruginosa* and *P. agardhii* grew in the presence of three non-AAs, i.e., Phe-Az, Prop-Lys, Prop-Tyr, in particular the Phe-Az addition reduced growth, increased cell size and reduced AF when compared with controls (i.e., cells grown in the absence of non-AAs but otherwise treated identically)^27,28^. For *P. agardhii* a significant decrease in AF was related to a very low A488-click signal which did not differ from controls^28^.

**Fig. 2:**
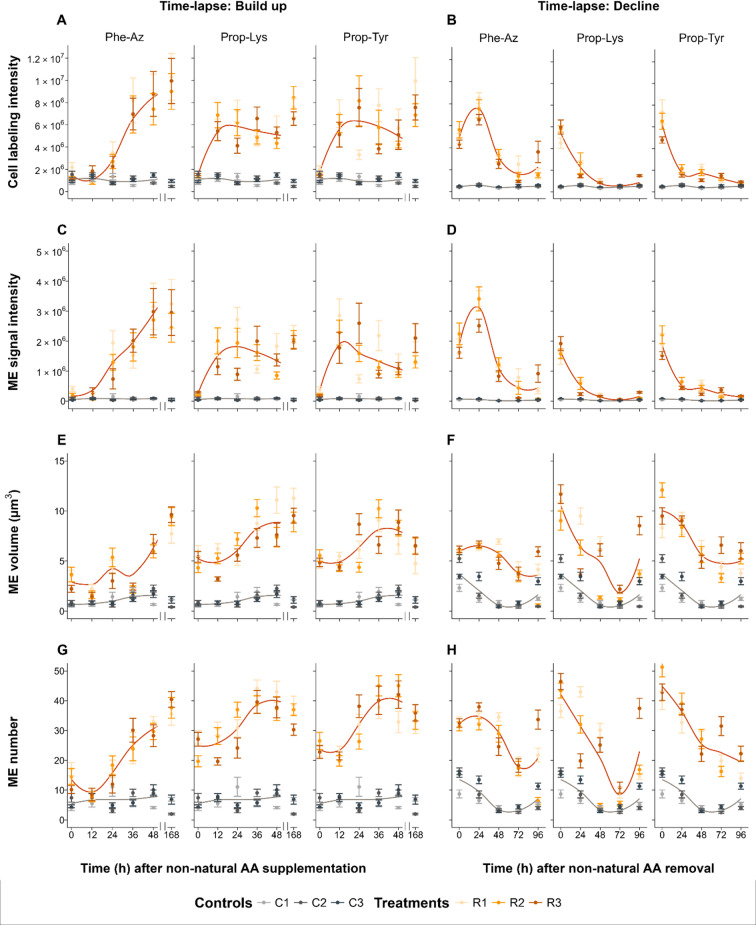
Visualization and quantification of cyanotoxin synthesis in *M. aeruginosa* strain Hofbauer during time-lapse experiments for build-up (**A**, **C**, **E**,** G**) or decline (**B**, **D**, **F**,** H**) as revealed by (**A**, **B**) labeling of clickable MCs via pulsed feeding of three non-AAs, i.e., Phe-Az, Prop-Lys and Prop-Tyr (orange). Controls (black) were grown and processed under identical conditions but without non-AA substrate. Advanced imaging analysis resulted in so-called model entities (ME), allowing the quantification of (**C**, **D**) total ME signal intensity (MEi), (**E**, **F**) total ME volume (MEv in µm^3^), and (**G**,** H**) ME numbers (MEn). Symbols represent mean ± SE. Regression lines were fitted following the LOESS method for all three replicates per time point (unless otherwise specified).

**Fig. 3:**
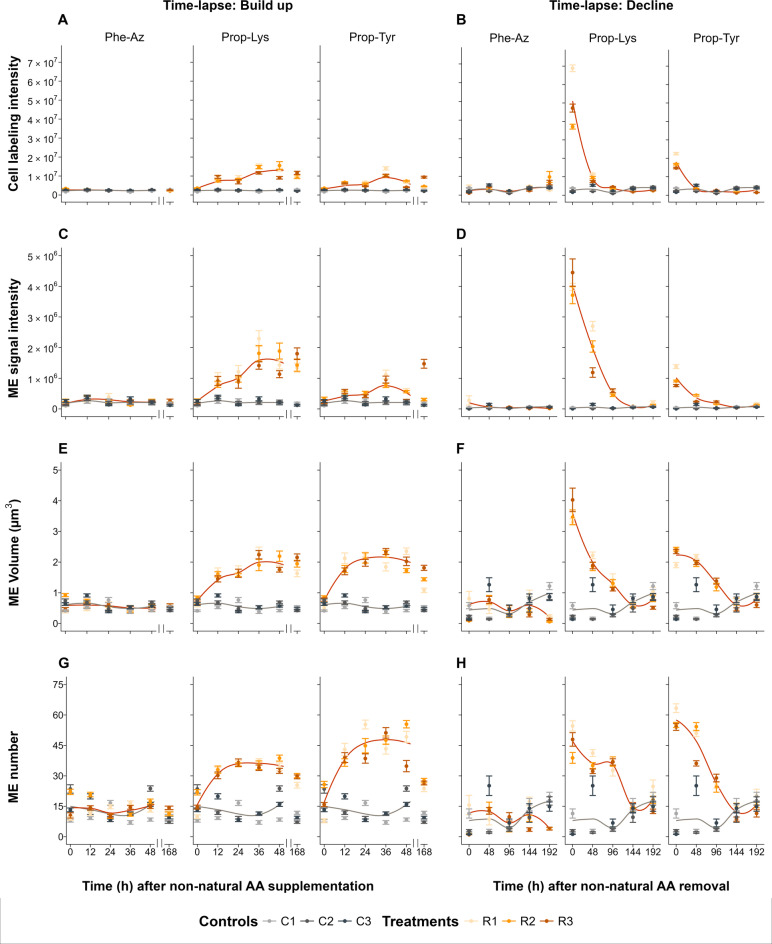
Visualization and quantification of cyanotoxin synthesis (mean ± SE) in *P. agardhii* strain No371/1 during time-lapse experiments for build-up (**A**, **C**, **E**,** G**) or decline (**B**, **D**, **F**,** H**) as revealed by (**A**, **B**) labeling of clickable APs via pulsed feeding of three non-AAs, i.e., Phe-Az, Prop-Lys and Prop-Tyr (orange). Controls (black) were grown and processed under identical conditions but without non-AA substrate. Advanced imaging analysis resulted in so-called model entities (ME), allowing the quantification of (**C**, **D**) total ME signal intensity (MEi) and (**E**, **F**) total ME volume (MEv in µm^3^), and (**G**,** H**) ME numbers (MEn).

During the *M. aeruginosa* signal build-up, cellular signal intensity rose rapidly within 12 h, with increases of 4.3-fold for Prop-Lys and 3.7-fold for Prop-Tyr-fed cells, stabilizing thereafter. Conversely, Phe-Az-fed cells exhibited a delayed signal increase, peaking at 5.7-fold after 48 h. The decline phase showed a rapid decrease by 2.4-fold for Prop-Lys and 3.5-fold for Prop-Tyr-fed cells within the first 24 h, while Phe-Az-fed cells decreased by 3.1-fold after 72 h (Fig. 2A, B). Similarly, cellular signal intensities for *P. agardhii* increased significantly in Prop-Lys and Prop-Tyr-fed cells, reaching a maximum of 4.6-fold and 4.0-fold after 36 h, respectively. In contrast, no increase was detected in Phe-Az-fed cells. During the time-lapse signal decline, the cellular signal intensities decreased sharply by 5.4-fold and 5.3-fold in the first 48 h for Prop-Lys-fed and Prop-Tyr-fed cells, respectively. As for the signal build-up experiment, no change was detected in Phe-Az-fed cells (Fig. 3A, B).

### Quantifying signal intensity in modeled entities from clickable cyanotoxins

**Fig. 4:**
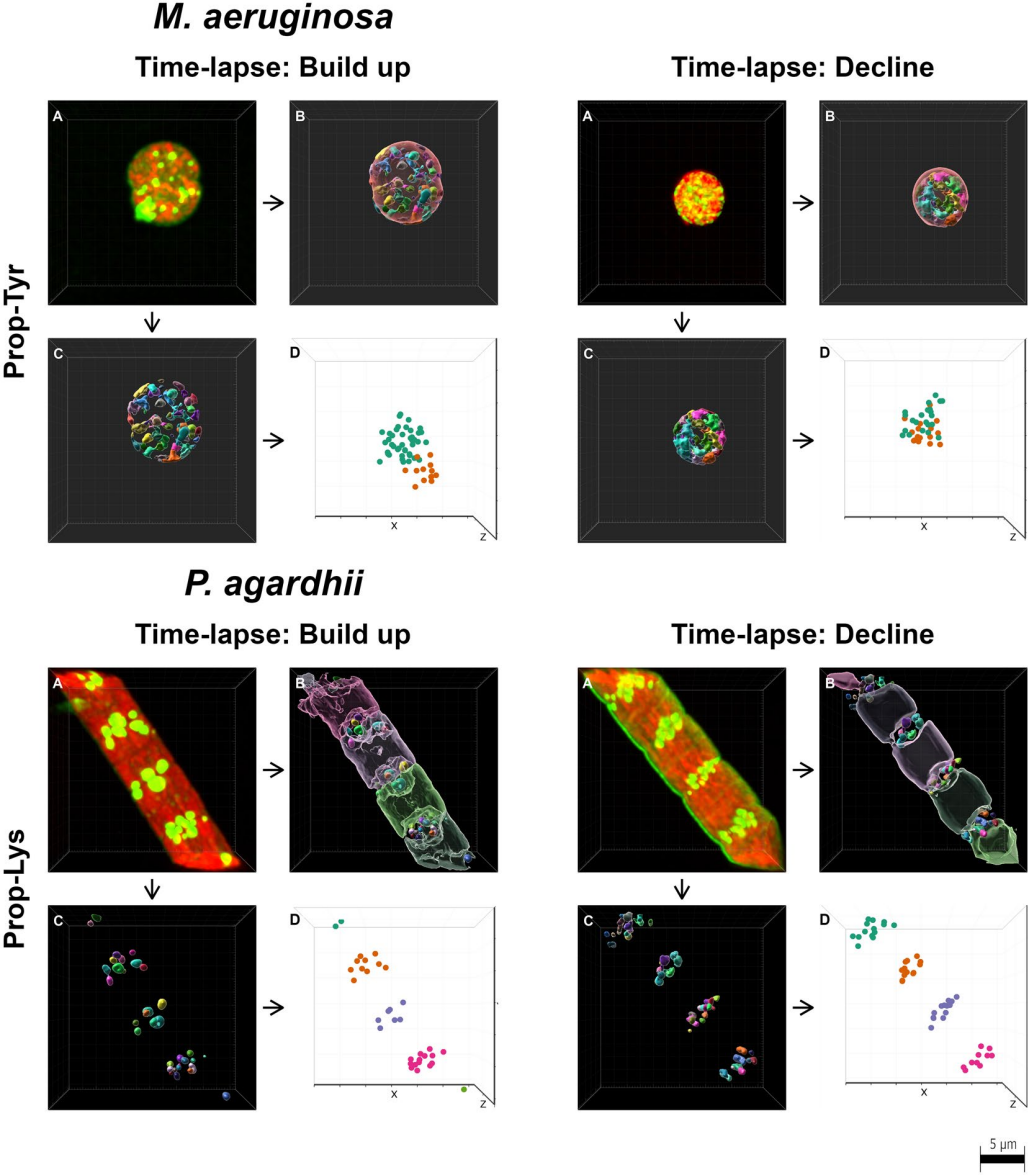
(**A**) Workflow for processing high-resolution 3D microscopy images of *M. aeruginosa* strain Hofbauer grown in the presence of Prop-Tyr to produce clickable MCs and *P. agardhii* strain No371/1 grown in the presence of Prop-Lys to produce clickable APs. (**B**) Autofluorescence of cells (red signal) was used to estimate the cell shape via Imaris surface function (1) (**C**) The A488-click signal (in green) was used to calculate modeled entities (ME) using Imaris surface function (2) (**D**) Clustering analysis of the ME (x, y, z) coordinates was performed to quantify the intracellular ME distribution. The analogous workflows for other non-AAs are shown in Extended Data Figs. 2 and 3.

As reported earlier, clickable MCs in *M. aeruginosa* and clickable APs in *P. agardhii* were identified as distinct entities^13,14,28^. Advanced imaging analysis resulted in so-called modeled entities (ME), allowing the quantification of intracellular distribution of ME-specific signal intensities (MEi), ME volumes (MEv), and ME numbers (MEn). (Supplementary Tables 1–3) while the cellular shape volume was estimated from AF. The workflow for processing images is shown in Fig. 4 (Extended Data Figs. 2 and 3).

For *M. aeruginosa*, the MEi constituted a significant fraction of the recorded total cellular signal intensity (mean ± SE, 7.3 ± 1.0–40.6 ± 1.2%), whereas in *P. agardhii*, MEi represented a smaller fraction (3.1 ± 0.3–19.7 ± 2.2%), (Extended data Figs. 4 and 5). Trends in MEi correlated with observed signal intensities: In *M. aeruginosa*, high correlations occurred during signal build-up (R^2^ ≥ 0.71) and during decline (R^2^ ≥ 0.70). *P. agardhii* showed high correlations for non-AA Prop-Lys and Prop-Tyr-fed cells during build-up (R^2^ ≥ 0.70) and decline (R^2^ ≥ 0.80), whereas the lowest correlations occurred for Phe-Az-fed cells (Extended Data Figs. 6 and 7).

**Fig. 5:**
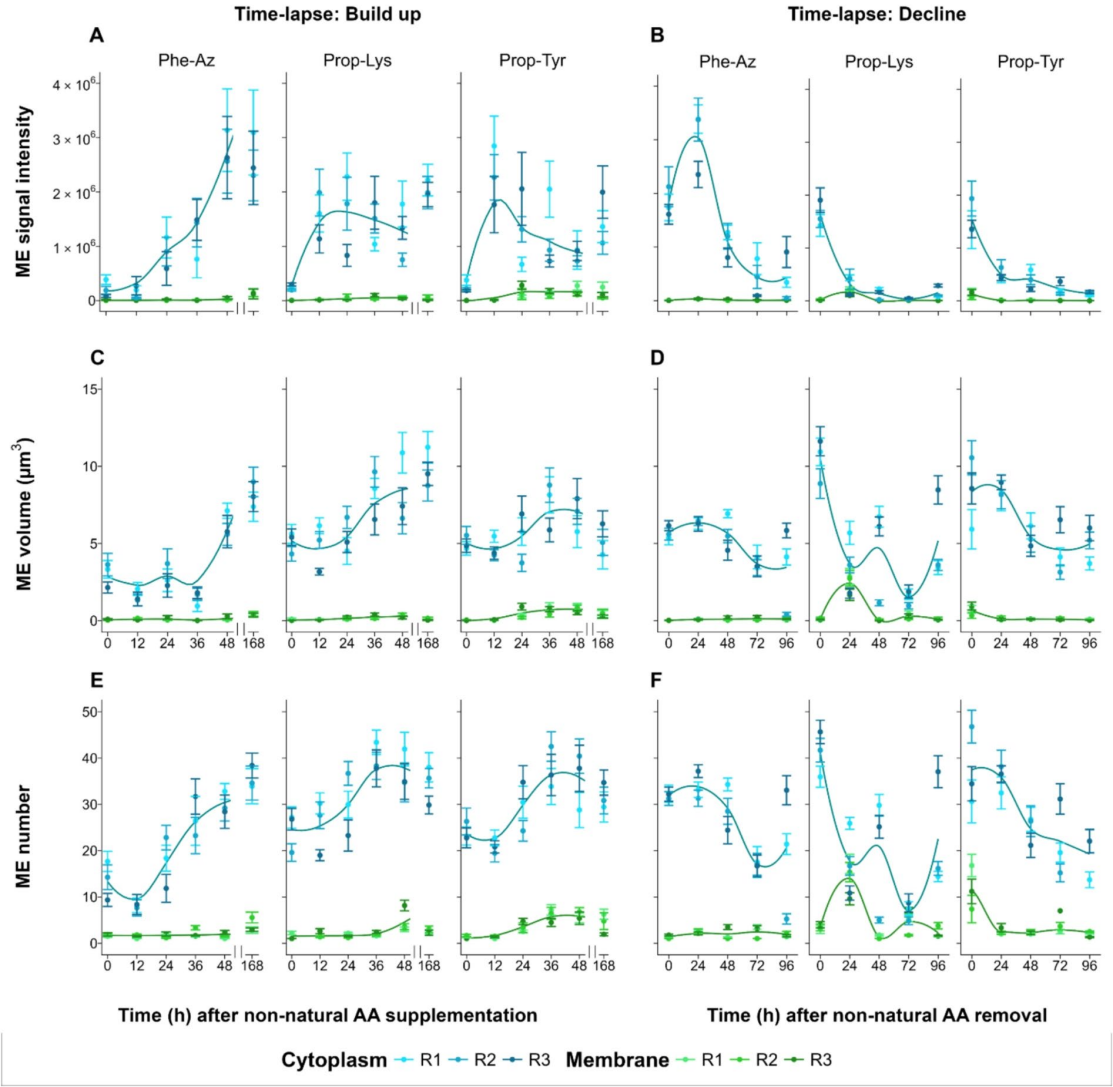
Compartmentation of cyanotoxin synthesis (mean ± SE) in *M. aeruginosa* strain Hofbauer during time-lapse experiments for build-up (**A**, **C**, **E**) or decline (**B**, **D**, **F**) as revealed by labeling of clickable MCs and advanced imaging analysis. (**A**, **B**) ME-specific signal intensity (MEi), (**C**, **D**) ME volume (MEv) and (**E**,** F**) ME numbers in cytoplasm vs. membrane region using the pulsed feeding of non-AAs (Phe-Az, Prop-Lys and Prop-Tyr) for clickable MCs production. Subcellular compartments were assigned according to the relative position of ME in the cell, i.e., in the cellular cytoplasm (blue; rel. pos. < 1) or in the membrane region (green; rel. pos. ≥ 1).

**Fig. 6:**
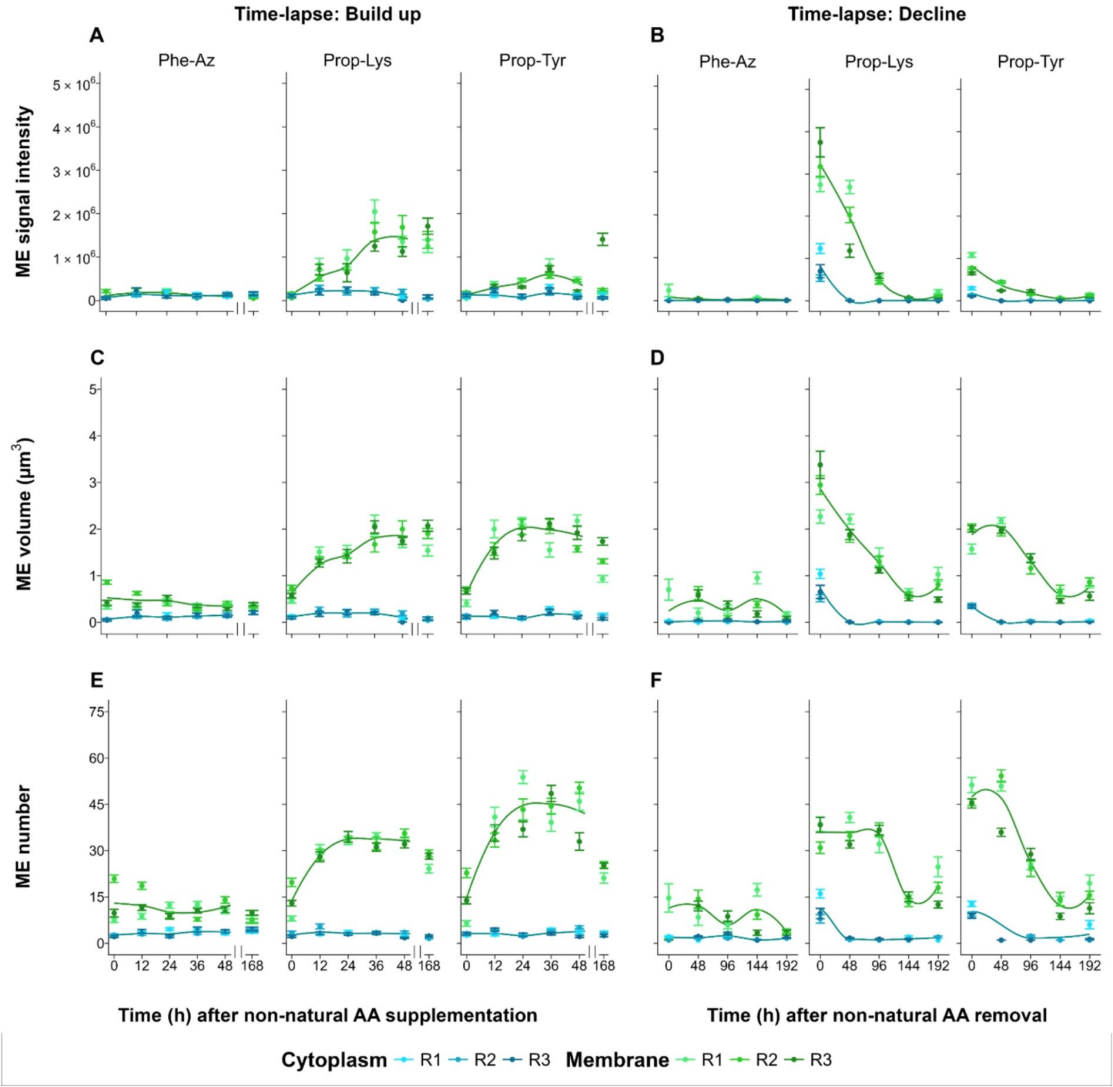
Compartmentation of cyanotoxin synthesis (mean ± SE) in *P. agardhii* strain No371/1 during time-lapse experiments for build-up (**A**, **C**, **E**) or decline (**B**, **D**, **F**) as revealed by labeling of clickable APs and advanced imaging analysis. (**A**, **B**) ME-specific signal intensity (MEi), (**C**, **D**) ME volume (MEv) and (**E**,** F**) ME numbers in cytoplasm vs. membrane region using the pulsed feeding of non-AAs (Phe-Az, Prop-Lys and Prop-Tyr) for clickable APs production. Subcellular compartments were assigned according to the relative position of ME in the cell, i.e., in the cellular cytoplasm (blue; rel. pos. < 1) or in the membrane region (green; rel. pos. ≥ 1).

**Fig. 7:**
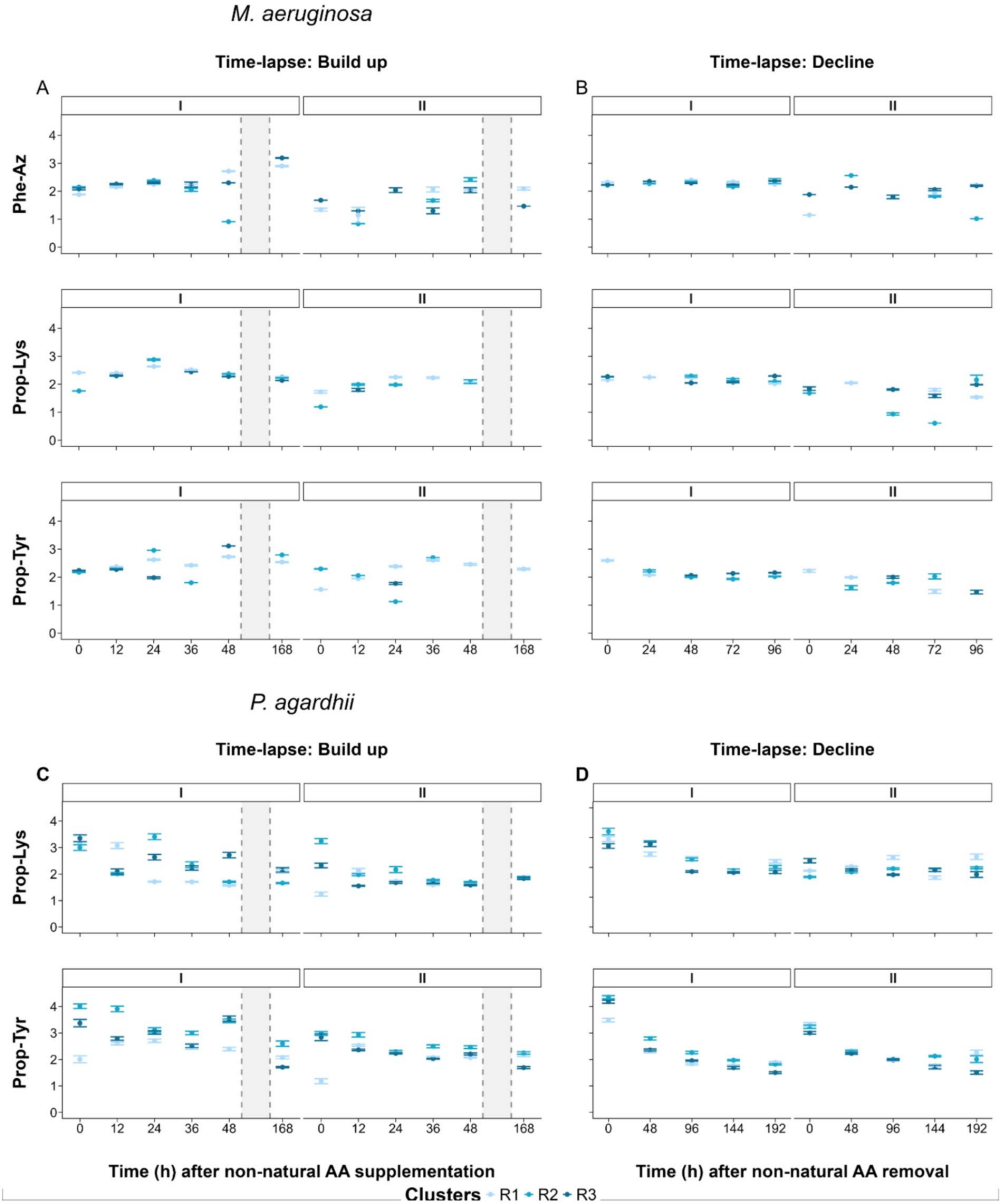
Quantification of ME distances (mean ± SE) within clusters in *M. aeruginosa* strain Hofbauer (**A**, **B**) and *P. agardhii* strain No371/1 (**C**, **D**) during time-lapse experiments for build-up (**A**,** C**) or decline (**B**,** D**) using the pulsed feeding of non-AAs (Phe-Az, Prop-Lys and Prop-Tyr) for clickable MCs/APs production. Note: Phe-Az fed cells of *P. agardhii* revealed non-detectable ME when compared with controls.

### Quantifying the volumes and numbers of modeled entities

To investigate the variation in MEi, we compared MEv and MEn in cells. For *M. aeruginosa*, during time-lapse build-up, MEv (µm^3^) increased up to 2.9–fold for Phe-Az, 1.9–fold for Prop-Lys and 1.7–fold for Prop-Tyr-fed cells, respectively. While during time-lapse decline MEv decreased down to 0.6–fold for Phe-Az and 0.5–fold for Prop-Lys and Prop-Tyr (Fig. 2C-F). Thus, MEv accounted for 4.0 ± 0.7–22.6 ± 1.7% of cellular shape volume during build-up and between 4.5 ± 0.6–27.7 ± 1.8% of cell shape volume during decline.

In comparison, the absolute MEv of *P. agardhii* were smaller and did not significantly differ from the controls for Phe-Az-fed cells (0.9–fold), which corresponds to the observed lowest signal intensity and MEi (Fig. 3C- F). During build-up, for both Prop-Lys and Prop-Tyr-fed cells the MEv increased 2.4–fold, representing a lower fraction of the filament shape volume (0.6 ± 0.1–1.6 ± 0.1%). During signal decline, MEv decreased 0.5–fold and 0.6–fold for Prop-Lys, and Prop-Tyr-fed cells, respectively, while accounting between 0.5 ± 0.1–2.4 ± 0% of the filament shape volume. Phe-Az fed cells of *P. agardhii* revealed non-detectable MEv when compared with controls.

Notably, during both build-up and decline experiments, the number of ME per cell or filament remained relatively stable (Supplementary Table 3). For *M. aeruginosa*, MEn increased 2.7–fold, 1.6–fold and 1.7–fold during build-up and decreased between 0.6–fold, 0.2–fold and 0.4–fold during decline for Phe-Az, Prop-Lys and Prop-Tyr-fed cells, respectively (Fig. 2G, H). Similarto MEv in *P. agardhii* the MEn for Phe-Az-fed cells did not increase during build-up and increased 2.0–fold and 2.2–fold for Prop-Lys and Prop-Tyr-fed cells, respectively. During decline MEn decreased 0.6–fold for Prop-Lys-fed and Prop-Tyr-fed cells, while for Phe-Az-fed cells no significant difference in MEn was observed (Fig. 3G, H).

In summary, both cyanobacteria exhibited significant variability in MEi, with *M. aeruginosa* showing a 4–13-fold increase and a 1–36-fold decrease, while *P. agardhii* had a 4–9-fold increase and a 1–50-fold decrease during build-up and decline, respectively (Supplementary Table 4). In contrast, MEv was less variable increasing 1–3-fold and decreasing 1–6-fold for both cyanobacteria (Supplementary Table 5). MEn demonstrated lower variation across both time-lapse experiments (Supplementary Table 6). Thus, the primary cause for changes in MEi was attributed to signal intensity variations, with MEv or MEn contributing to a lesser extent.

### Subcellular localization of modeled entities

The subcellular localization of ME was quantified in relation to cell shapes modeled from AF. For *M. aeruginosa*, modeled cell shape volumes closely matched measured volumes during both time-lapse experiments. For *P. agardhii*, the modeled volumes accounted for about 50% of the measured filament volume (Extended Data Figs. 8 and 9). In order to minimize the influence of the cell shape volume on ME localization the ME were classified according to their relative positions in the cell and assigned to the membrane region (relative position > 1) or to the cytoplasmatic region (rel. pos. ≤= 1).

Overall, the subcellular localization of ME in *M. aeruginosa* remained stable during both time-lapse experiments, with positions ranging from 0.8 to 0.9 of the cellular radii, indicating a cytoplasmic location (Supplementary Table 7). In contrast, ME in *P. agardhii* were predominantly located near the cell periphery, ranging from 1.1 to 1.3 of the cell radii during both time-lapse experiments (Extended Data Figs. 10 and 11).

### Quantifying the subcellular distribution of modeled entity intensities and volume

According to the relative ME positions for *M. aeruginosa*, most MEi and MEv were predominantly found in the cytoplasm during signal build-up (Fig. 5A-D), i.e., accounting for 79.8 ± 5.5% to 99.2 ± 0.1% of all MEi and 79.9 ± 3.3% to 99.4 ± 0.1% of all MEv. In contrast, those ME near the membrane accounted for 0.8 ± 0.1% to 20.2 ± 5.5% of MEi and 0.6 ± 0.1% to 20.1 ± 3.3% of cellular volume (Extended Data Fig. 12). During decline, cytoplasmic ME continued to be most abundant, i.e., making up 58.9 ± 4.8% to 99.6 ± 0.2% of total MEi and 79.9 ± 3.3% to 99.4 ± 0.1% of MEv. The membrane ME accounted for much lower proportions (0.4 ± 0.2% to 41.1 ± 4.8% of all MEi and 0.4 ± 0.1% to 41.8 ± 4.9% of all MEv).

For *P. agardhii* the opposite subcellular distribution was observed (Fig. 6A-D), i.e., during signal build-up MEi in membrane region accounted for 62.5 ± 5.9% to 95.2 ± 1.4% of total MEi and 83.1 ± 0.8% to 96.0 ± 1.2% of MEv (Extended Data Fig. 13). Conversely, cytoplasmic ME were significantly lower, accounting only for 4.8 ± 1.4% to 37.5 ± 5.9% of MEi and 4.1 ± 1.2% to 16.9 ± 0.8% of MEv. Similarly, during time-lapse decline the ME in the membrane represented larger proportions of MEi (80.0 ± 5.8–99.9 ± 0.1%) and MEv (79.5 ± 5.7–99.8 ± 0.1%), when compared with MEi (0.1 ± 0.1–20.0 ± 5.8%) and MEv (0.2 ± 0.1–20.5 ± 5.7%) in the cytoplasm.

Proportions of MEn in the two compartments followed an analogous pattern. For *M. aeruginosa* during signal build-up, MEn located in the cytoplasm accounted for the vast majority (Fig. 5E, F), i.e., 85.1 ± 1.4% to 99.1 ± 0.2% while those in the membrane accounted for 7.0 ± 1.5% to 51.7 ± 4.3%. Similarly, during time-lapse decline cytoplasmatic MEn were dominant and ranged from 54.9 ± 3.5% to 99.0 ± 0.3%. While those near the membrane contributed a minor share, i.e. 4.3 ± 1.1% to 58.7 ± 16.7%. For *P. agardhii* in contrast, membrane MEn were much higher (Fig. 6E, F) accounting for 66.1 ± 1.7% to 96.4 ± 0.8% during signal build-up and 77.2 ± 3.4% to 99.8 ± 0.2% during decline. Whereas those in the cytoplasm region ranged from 5.8 ± 1.4 to 21.3 ± 5.1% during signal build-up and 2.0 ± 0.4% to 22.8 ± 3.4% during decline. Not surprisingly Phe-Az fed cells of *P. agardhii* revealed lowest numbers of MEn.

### Quantifying the distances between modeled entities

To investigate the subcellular localization of ME independent of cell shape, a clustering analysis was carried out using ME position coordinates. In *M. aeruginosa*, 85.8 ± 2.4% of ME were assigned to cluster I and only 11.4 ± 2.0% to cluster II. A third cluster III was rarely detected in Phe-Az and Prop-Tyr-fed cells. In *P. agardhii*, up to six clusters (I-VI) were identified for Prop-Lys or Prop-Tyr-fed cells, with four major clusters comprising between 34.9 ± 1.8–11.7 ± 1.4% of total ME, consistently located near the septa of cells in the trichome.

For *M. aeruginosa*, the ME distances within the two major clusters for Phe-Az, Prop-Lys and Prop-Tyr-fed cells were similar during signal build-up (I: 3.05 ± 0.14–1.98 ± 0.55; II: 2.65 ± 0.05–1.09 ± 0.14) and signal decline (I: 2.35 ± 0.03–2.00 ± 0.07; II: 2.36 ± 0.21–1.33 ± 0.37), (Fig. 7A, B). Similarly, for *P. agardhii* the ME distances within clusters were comparable during signal build-up (I: 3.17 ± 0.10–1.97 ± 0.16; II: 2.61 ± 0.17– 1.65 ± 0.03) and during signal decline (I: 4.0 ± 0.27–1.74 ± 0.12, II: 3.19 ± 0.09–1.83 ± 0.09), (Fig. 7C, D). Overall, *M. aeruginosa* showed rather stable ME distances, while those of *P. agardhii* demonstrated higher variability.

Since *M. aeruginosa* typically grew in single cells while *P. agardhii* grew in filaments it was interesting to know whether dividing cells of *M. aeruginosa* might differ in ME subcellular distribution. Thus, a number of dividing cells (*n* = 92 during time-lapse build-up or n = 72 during decline) co-occurring with single cells were compared in ME within cluster distances as well as compartmentation. More than one ME cluster was more frequently detected in dividing cells for Phe-Az (66.7% vs. 38.2%, dividing vs. single cells), but not for Prop-Lys (17.7% vs. 17.2%) and Prop-Tyr (28.6% vs. 27.1%) cells during time-lapse build-up. Contrastingly, during time-lapse decline dividing Phe-Az-fed, Prop-Lys-fed and Prop-Tyr-fed cells frequently presented more than one ME cluster (58.6% vs. 15.2%; 54.5% vs. 31.0%; 44.4% vs. 28.1%; respectively). Overall, dividing cells did not differ from single cells in their ME localization. For example, in cytoplasm ME relative positions ranged 0.73–0.90 vs. 0.65–0.90 and 0.75–0.89 vs. 0.68–0.89 in single cells vs. dividing cells during time-lapse build-up and decline, respectively. Similarly, in membrane region ME relative positions varied narrowly between 1.07 – 1.21 vs. 1.05–1.22 and 1.08–1.23 vs. 1.06–1.21 during time-lapse build-up and decline, respectively (Extended Data Fig. 14).

## Discussion

In this study, we quantified toxin/peptide synthesis in 3D and monitored subcellular compartmentation during time-lapse experiments. For *M. aeruginosa* and *P. agardhii*, the MEi per cell explained a significant fraction of the recorded total signal intensity per cell, i.e., 7.3 ± 1.0–40.6 ± 1.2% and 3.1 ± 0.3–19.7 ± 2.2%, respectively. Signal intensities changed sharply (Figs. 2 and 3), while the MEv, MEn and intracellular distribution of ME remained relatively stable. Notably, the localization of ME differed between the two genera. In *M. aeruginosa*, ME located closer to the cell centroid, whereas in *P. agardhii* ME located near the cell periphery.

### Combination of click-chemistry labeling with the immunolabeling of MC

Standard immunofluorescence labeling was applied to visualize cellular structures or compartments in cyanobacteria, employing specific antibodies such as anti-RbcL (Rubisco large subunit, form I and form II) and anti-FtsZ (procaryotic cell division GTPase) for the cytoplasm in cyanobacteria or anti-PsbA (D1 protein of PSII, C-terminal) as a thylakoid membrane marker^13^. A double-labeling protocol was established to label clickable MCs and protein, revealing increased intracellular spatial correlation between PsbA and AF, but the same was not observed between clickable MCs and immunolabeled proteins^13^. In this study, a similar protocol was applied in *M. aeruginosa* using two fluorophores (A555-azide/alkyne and sAB-A488). It was hypothesized that the correlation between click-labeled MCs and immunolabeled MCs would be greater than those between either group and the AF. Results confirmed that a significant fraction of the intracellular MC pool can be identified by either click-chemistry or immunolabeling (Fig. 1). For other bioactive peptides, such as APs, the generation of antibodies has not been successful because of cross-reactivity with other peptide families^30^. It is possible that the high structural diversity among peptide families and the scarcity of unique moieties (such as the Adda side chain in MC) limit the feasibility of developing specific antibodies for certain peptide families. Thus, chemoselective labeling offers a viable alternative for visualizing these peptides.

### Differentiating the intracellular origin of cyanotoxin biosynthesis

Hypotheses explaining the observed heterogeneity in signal intensity include facilitating intracellular accumulation, ongoing molecular interactions, and places of cyanotoxin biosynthesis. It has been argued that intracellular MC/AP contents may exceed solubility thresholds, with concentrations in the femtogram to picogram range, closely related to chlorophyll a contents^19,31^. Thus, abundant MCs and bioactive peptides may participate in metabolic processes such as carbon/nitrogen metabolism, antioxidation, colony formation, and intercellular communication^7^. To date, various mutually nonexclusive hypotheses have been proposed, such as MC-assisted carbon acquisition^32^ and/or the protection of cells and metabolic processes from photooxidation and oxidative stress^33,34^. On the one hand, MCs themselves and other bioactive peptides^35^ can serve as antioxidants under oxidative stress conditions^36^. On the other hand, MCs have been shown to interact with specific proteins, such as Rubisco, even more so under high light conditions or under H_2_O_2_ addition or iron-limited conditions^7,29^. In this study, MEi increased significantly (6.46 ± 0.89 to 12.52 ± 1.01-fold), while MEv (1.18 ± 0.12 to 2.85 ± 0.19-fold) and MEn (1.41 ± 0.04 to 2.72 ± 0.10-fold) exhibited more moderate increases in *M. aeruginosa* over 168 h. These findings suggest that ME were characterized by average intracellular stability, potentially indicating the origins of MC/AP biosynthesis within the cell.

Although the biosynthetic pathways for MCs and APs are known^9,10^, their subcellular localization remains unclear. Analogously numerous natural compounds (bioactive peptides) are produced by NRPS-PKS complexes across various prokaryotic phyla and Ascomycota fungi^37^. Studies, such as from Ishikawa et al.^38^, have used a chemical probe directed to the active sites of NRPS domains to measure the activity of surfactin-producing NRPS in *Bacillus subtilis*. Additionally, the immune localization in transmission electron microscopy (TEM) of PKS multienzymes synthesizing bacillaene in *B. subtilis* revealed a heterologous subcellular distribution^39^. For *Pseudomonas aeruginosa*, the pyoverdine NRPS is associated with membranes^40^, and recent work highlights the assembly of pyoverdine NRPS transmitting from cytoplasm to periplasm^41^. Since pyoverdine has been reported to act as a siderophore in iron uptake the potential membrane-bound organization of pyoverdine NRPS has been postulated under the name “siderosomes”^42^. Hence, a similar compartmentation of NRPS complexes may occur in cyanobacteria that might be related to biological function (see below) or the efficiency of biosynthesis^43^.

### Modeled entity compartmentation and subcellular distance

An important question for localization is whether toxin synthesis takes place in a specific cellular compartment and whether the synthesized peptides are stored in compartments within cells. In general, cyanobacterial cells are organized into three different compartments formed by the three membranes (outer membrane, cell membrane, and thylakoid membrane), resulting in periplasmatic space, cytoplasm, and thylakoid lumen. Seminal immunogold labeling studies indicate that MCs predominantly localize in the thylakoid area^44,45^. In this study, individual cells were divided into two compartments, namely, the cytoplasmic region and the membrane region, and their ME-specific characteristics were compared. For *M. aeruginosa*, much lower MEi, MEv and MEn were observed in the membrane region than in the cytoplasm (Fig. 5). In contrast, for *P. agardhii*, the share of MEi, MEv and MEn in the membrane region was significantly greater than that in the inner cell (Fig. 6). These results align with the general observation that ME in *P. agardhii* are located more closely along the septa between cells.

Clustering analysis of ME indicated consistent clusters in *P. agardhii*, located near the septum between cells in the trichome. Regardless of the differences in location, both *M. aeruginosa* and *P. agardhii* exhibited similar distances between ME within clusters, suggesting comparable aggregation factors for NRPS synthesis clusters (Fig. 7). Notably in *P. agardhii* distances between ME clusters were akin to the length of individual cells^46^. Conversely, most ME in *M. aeruginosa* were grouped into a single cluster, with a second cluster occurring in possibly dividing cells (Extended Data Fig. 14).

cHAB-forming cyanobacteria can be regulated by infections from various antagonistic organisms, including bacteria or protozoans like chytrids (Chytridiomycota)^47–49^. Chytrids typically infect the ends of *Planktothrix* trichomes^50,51^. This infection process compromises trichome integrity and increases vulnerability to grazing^52^. Hence, locating AP synthesis centers in the septa region may provide a selective advantage, hindering the propagation of rhizoid infection from cell to cell^53^. In contrast, *M. aeruginosa* grows unicellularly in mucilage^54^, and each cell needs to be individually infected or grazed by chytrid fungi or other antagonistic microbes^55,56^. Thus, differences in the localization of peptide synthesis might exist between cyanobacteria, which are related to morphological traits such as filament formation.

### Implications of cyanotoxin biosynthesis compartmentation

In general MC or AP chemoselective signals suggest the existence of distinct cyanotoxin biosynthesis centers in cells, differing in their localization between unicellular and multicellular cyanobacteria. Perhaps the strongest independent support for the working hypothesis investigated during this study comes from alternative approaches aiming to probe MC synthesis using well known catalyzed reporter deposition fluorescent in situ hybridization (CARD-FISH) via the MCYA probe^57,58^. Alike the chemoselective labeling, the strength of CARD-FISH is to visualize and quantify the heterogeneity of physiological processes in the individual cell^59^. The authors describe MC synthesis via mRNA of the *mcy*A gene resulting in non-uniform labeling dots spread within the cytoplasm and densely packed cytoplasmic labeling^58,60^. Correspondingly Zeller et al.^57^ observed under variable light exposure that the fluorescence intensity increased more than the number of spots implying locally increased transcription of the *mcy*A gene. Thus, combining CARD-FISH based visualization of *mcy*A gene expression with chemoselective labeling of MC would allow to quantify regulative effects on NRPS synthesis including transcription till the peptide product at the single cell level.

In the future, by using chemoselective labeling it will become possible to quantify intracellular and extracellular fluxes of clickable peptides by comparing signal intensities in MEs under experimental conditions. For example, for MCs functional roles such as participating in carbon/nitrogen metabolism, antioxidative processes, morphological characteristics (colony formation), etc. have been postulated^7^. Using chemoselective labeling, the flux of clickable MC or AP can be quantified under physiological stress conditions. For example, under high light exposure clickable MC is expected to bind to Rubisco and other abundant proteins because of intracellularly increased pH resulting from photosynthesis^29,61,62^. Remarkably MC structural variants in *M. aeruginosa* typically contain an alpha, beta-unsaturated carbonyl group in position 7 of the MC molecule which is reactive towards thiols under increased pH^63,64^. Thus, comparing the variation in signal intensity from MEs and other intracellular structures will inform about chemical interactions and allow to discriminate between mutually non-exclusive physiological roles.

## Materials and methods

In this study, the application of click-chemistry reactions allowed us to track and investigate clickable MC/AP synthesis under reduced invasive conditions through confocal microscopy and advanced microscopy imaging analysis. Recent developments in machine learning (ML) algorithms have enabled the automated annotation of subcellular structures and hence the processing and analysis of previously unattainable large numbers of images. In total, 2642 individual *M. aeruginosa* cells, from which 164 (6.2%) were undergoing cellular division and 2606 *P. agardhii* filaments were investigated.

### Study organisms

*M. aeruginosa* strain Hofbauer (isolated from Lake Neusiedl, Niederösterreich, Austria, by Barbara Hofbauer in 1982) and *P. agardhii* strain No371/1 (isolated from Moose Lake, Alberta, Canada, by Rainer Kurmayer in 2005) were grown in BG11 medium^65^ at 20 °C using 50 µmol photons m^− 2^ s^− 1^ (16:8 h light/dark cycle) of irradiation^19^. Both strains have been shown to carry a promiscuous A-domain of specific NRPS of either MC or AP synthesis, resulting in co-occurring MC or AP structural variants, i.e., MCs with the exchange of Tyr vs. Leu in pos. 2^11^ or APs with the exchange of Arg vs. Tyr vs. Phe in pos. 1^16^. With respect to AP biosynthesis, the first A-domain, ApnAA_1,_ was previously heterologously expressed and crystallized, elucidating the structural basis of this promiscuous activity^12^. This promiscuous amino acid incorporation has been successfully exploited to produce clickable APs and conduct subsequent labeling via CuAAC reactions^13,14^. Analysis of peptide extracts using LC-MS revealed no other peptide modifications^13,27^.

### Time-lapse experiments under maximum growth rate conditions

**Fig. 8:**
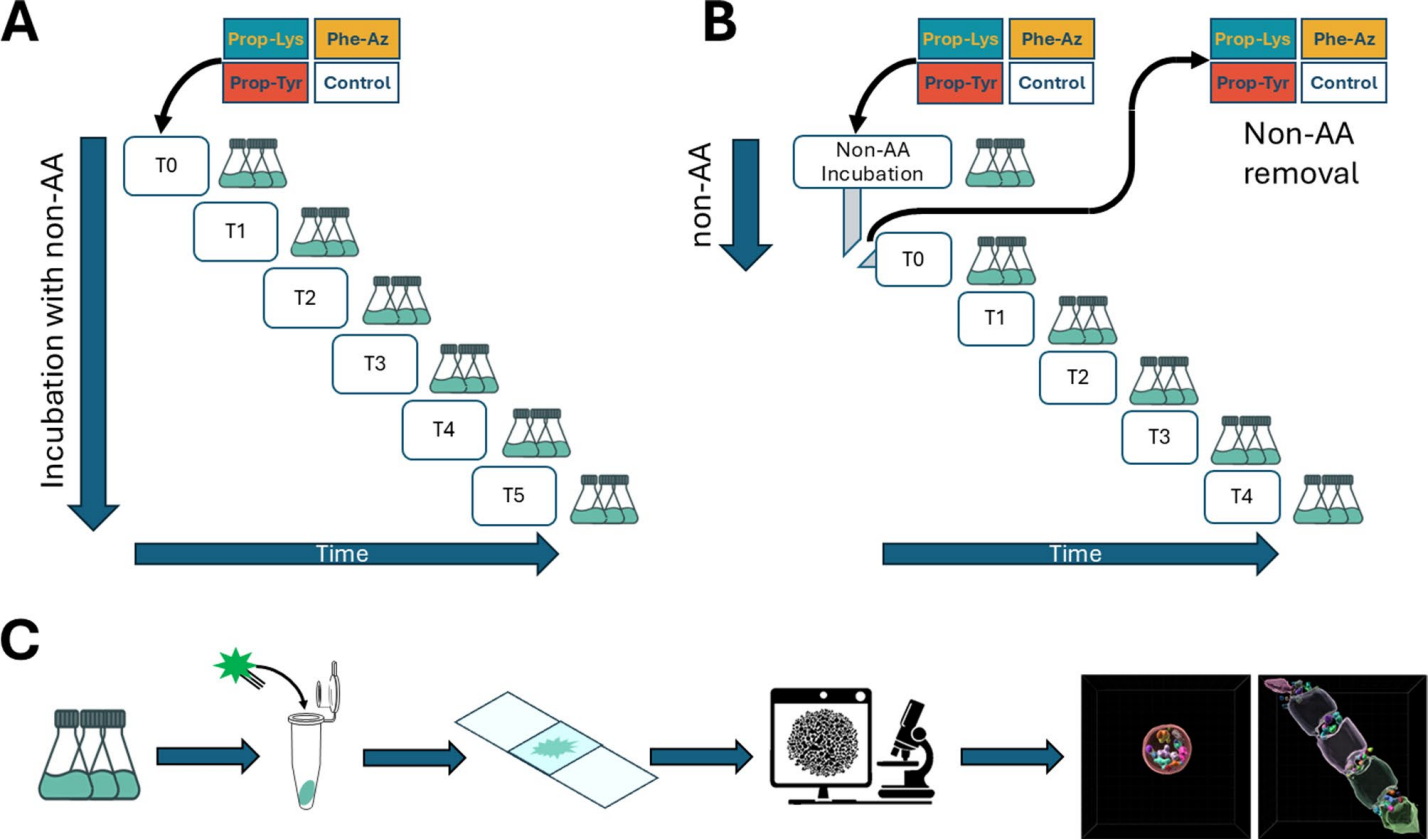
Workflow representing the experimental design for the time-lapse experiments. For time-lapse build-up (**A**), one specific non-AA (Phe-Az, Prop-Lys or Prop-Tyr) was supplemented into the cultures. Control cultures were grown without non-AA substrate and treated identically. Directly after the addition of non-AA substrate to the media, cells were harvested within 1 h (T0). Subsequently cells were harvested every 12 h (12–48 h), with a final harvest at T5 (168 h). For time-lapse decline (**B**), cells were initially grown with one of the non-AA substrates (48 h). Thereafter, the cells were collected by centrifugation, and the cells were transferred to non-AA free media and harvested within 1 h (T0). Subsequently cells were harvested every 24 h for *M. aeruginosa* or every 48 h for *P. agardhii* (T1-T4). **(C)** After harvesting all cells were processed identically, i.e., fixed with PFA and labeled with A488-azide (Prop-Lys and Prop-Tyr) or A488-alkyne (control and Phe-Az) and microscopic slides were prepared. Confocal microscopy imaging was performed to record A488-click signal intensity and AF. Using advanced imaging analysis signals were quantified by calculating modeled entities in relation to cellular shape volume.

The following experiments have been described in detail previously^27,28^ and the experimental design is shown in Fig. 8. In brief, for time-lapse signal build-up, at T0, the medium was supplemented once with one non-AA, either 4-azidophenylalanine (Phe-Az; Carl Roth, Karlsruhe, Germany), or *N*-propargyloxy-carbonyl-L-lysine (Prop-Lys; Sichem, Bremen, Germany) or O-propargyl-L-Tyrosine (Prop-Tyr; Iris Biotech, Marktredwitz, Germany) to a final concentration of 0.05 mM. Control cells were grown in parallel under identical conditions but in the absence of non-AAs. At T0, the cells were harvested directly after the non-AA was added to the medium within 1 h. To observe the change in A488-click signal intensity, cultures were incubated in presence of non-AA substrate and harvested every 12 h for the first 48 h (T1–T4), with a final harvest at T5 (168 h). For time-lapse signal decline, cultures were grown for 48 h in the presence of one of the three non-AAs. At T0, following 48 h of non-AA feeding, the cultures were washed by centrifugation (4000 g, 5 min) and transferred to fresh (non-AA-free) BG11 medium. Analogous to the build-up experiment, at T0, the cells were harvested within 1 h after they were transferred to fresh medium. The time points T1–T4 represented every 24 h for *M. aeruginosa* and every 48 h for *P. agardhii*. Three technical replicates were used. All the harvesting steps were performed at room temperature.

The addition of Phe-Az reduced the growth rate significantly in *M. aeruginosa* and *P. agardhii* due to toxic side effects. Possibly the intracellular decomposition of Phe-Az resulted in the formation of reactive nitrenes^66^. Since AF decreased with Phe-Az as well^28^, it was assumed that reactive decomposition products counteracted fluorescence generation in general. Interestingly, *M. aeruginosa* was less sensitive to Phe-Az than *P. agardhii* despite showing clickable MC-Az and high intracellular Phe-Az content^28^.

### Incorporation efficiency of non-AAs into clickable peptides as determined by chemical analytical tools

The production of clickable MCs/APs was analyzed by HPLC-MS^n^, quantifying clickable peptides showing the predicted molecular masses [M + H]^+^ for MCs or APs, e.g., resulting in MC-Az, MC-Lys/Tyr-alkyne or AP-Az, AP-Lys/Tyr-alkyne^13,14,27^, and characteristic MS^n^ fragmentation patterns.

To characterize incorporation or turnover efficiency of non-AAs into clickable MCs their percentages (based on total MC) were determined using liquid chromatography-mass spectrometry (LC-MS) from aliquot samples. For *M. aeruginosa* percentages for clickable MC increased from 2.4% to 21.3%, 0 to 3.5% and 3.6% to 89.5% of total MC during signal build-up in Phe-Az-fed, Prop-Lys-fed and Prop-Tyr-fed cells, respectively. Vice versa clickable MC declined in proportion from 16.0% to 6.6%, 2.1% to 0.6%, 43.2% to 6.6% for Phe-Az, Prop-Lys and Prop-Tyr during signal decline^27^. Similarly for *P. agardhii*, during signal build-up the incorporation efficiencies of non-AAs into clickable APs (based on total AP) increased from 0 to 21.1%, 3.0% to 84.2%, and 0 to 5.6% for Phe-Az-fed, Prop-Lys-fed and Prop-Tyr-fed cells, respectively. Whereas clickable AP decreased from 17.6% to 1.7%, 58.2% to 47.3% and 4.3% to 0 for Phe-Az, Prop-Lys and Prop-Tyr during signal decline^27^.

### Cell fixation and labeling

This protocol has been described in detail previously^28^. In brief, cells were collected in sterile 50 ml Falcon^®^ tubes, pelleted by centrifugation (4000 rpm) at room temperature (RT) after breaking the gas vesicles via the hammer, cork and bottle method, washed with fresh phosphate-buffered saline (PBS), fixed for 15 min in 2% paraformaldehyde (PFA) in PBS and permeated with Triton X-100 (0.1%) in PBS for 1 min^13^.

The clickable MCs/APs were labeled using standard copper-catalyzed azide–alkyne cycloaddition (CuAAC)^67^ using either the green fluorophore ALEXA Fluor 488 azide (A488-azide, #A10266) or ALEXA Fluor 488 alkyne (A488-alkyne, #A10267), (Invitrogen, Thermo Fisher Scientific, Darmstadt, Germany). The cells were rinsed with PBS containing 2% bovine serum albumin (BSA) for 30 min, after which the reaction was incubated in the dark (60 min) following the manufacturer’s instructions (Click-it^®^ Cell Reaction Buffer Kit; Thermo Fisher Scientific, Darmstadt, Germany). Labeling reactions were conducted distinctively using 1 µM of either A488-azide for the Prop-Lys and Prop-Tyr-fed cells or 1 µM of A488-alkyne for the Phe-Az-fed cells and control cultures. Cells were distributed over cover slips (0.17 ± 0.01 mm; Glaswarenfabrik Karl Hecht, Sondheim vor der Rhön, Germany) and mounted using antifade solution (ProLong^®^ Diamond Antifade Mountant, Thermo Fisher Scientific, Darmstadt, Germany). Microscopy slides were stored refrigerated in the dark until analysis.

### Combination of click-chemistry labeling with immunolabeling of microcystins

To compare with the chemoselective labeling of clickable MCs in *M. aeruginosa* the widely used monoclonal primary antibody for MCs (monoclonal antibody against microcystin-LR (#MC10E7), Enzo Life Sciences, Switzerland^68^ coupled to goat anti-mouse IgG (H + L) superclonal™ secondary antibody Alexa Fluor 488 (#A28175) was used. Additionally, a polyclonal antibody (Agrisera AB, Vännä, Sweden) against Rubisco large subunit form I and form II (#AS03 037) linked to goat anti-rabbit IgG (H + L) superclonal secondary antibody Alexa Fluor 488 (#A27034) was used as a control for the performance of the immunolabeling reaction. For this purpose, a double-labeling protocol including firstly, the chemoselective labeling of clickable MCs via ALEXA Fluor 555 alkyne (A555-alkyne, #CLK-092-1, Jena Bioscience, Jena, Germany) or ALEXA Fluor 555 azide (A555-azide, #CLK-090-1) and secondly, immunolabeling of MC-LR or Rubisco was established^13^: *M. aeruginosa* cultures were harvested by centrifugation and washed in PBS as described above. In this protocol, the cells were fixed by incubation in 96% EtOH (-20 °C) for 15 min, subsequently washed with PBS and stored at 4°C^13,69^. The cells were first used for the CuAAC reaction using A555-azide/alkyne and subsequently for the immunolabeling reaction (sAB-A488). Subsequently, the cells were immobilized on cover slips, air-dried, blocked for 30 min with PBS (2% BSA) at RT, and incubated for 60 min at RT with the corresponding primary antibody (1:250 dilution in PBS 2% BSA). Following incubation, the cells were washed with fresh PBS, air-dried and incubated for 60 min at RT in the dark with secondary anti-mouse (MC) or anti-rabbit (RbcL) monoclonal antibodies carrying A488 fluorophores (sAB-A488; 1:100 in PBS supplemented with 2% BSA). Finally, the cells were washed (3×) with PBS and (1×) Millipore water, air-dried, mounted on microscopy slides and stored at 4 °C.

### Microscopy analysis

High-resolution images were obtained using an SP8 laser scanning confocal microscope (Leica Microsystems, Wetzlar, Germany) at the Biooptics Core Facility (CCB) maintained by Medicinal University and the University of Innsbruck and reported previously^28^. Microscopy images were taken with an XY resolution of 50 nm and a Z resolution of 150 nm. In total, 20 randomly selected individual cells or filaments were processed per technical replicate. During image acquisition, care was taken to prevent oversaturation conditions; i.e., the power of the white light laser (WLL) was adjusted prior to image acquisition.

The three-dimensional raw images were analyzed, including the deconvolution of raw images, determination of the region of interest (ROI) representing the cell/filament, and measurement of the signal intensities from ROIs using Huygens Essential 23.10 software (Scientific Volume Imaging, Hilversum, The Netherlands), as follows: The first channel (Ch0) measured the labeling signal intensity from either A488-azide/alkyne fluorophores (495 nm excitation, 500–550 nm emission) for clickable MC or sAB-A488 for MC immunodetection, and a second channel (Ch1) recorded the natural autofluorescence (AF) intensity from the cells (620 nm excitation, 680–730 nm emission). For immunolabeled cells, a third channel (Ch2) was used to measure signal intensity from A555 fluorophores (555 nm excitation, 560–610 nm emission) for clickable MCs.

The cellular shape volume was calculated from AF for both strains using a sphere (*M. aeruginosa* cells) or a cylinder (*P. agardhii* filaments) as approximated geometric shapes. For immunolabeling in *M. aeruginosa*, colocalization coefficients between the immunolabeled MC signal (sAB-A488) and the AF, between the click MC labeling signal (A555-click) and the AF, and between clicked MCs (A555-click) and immunolabeled MCs (sAB-A488) were calculated using Huygens Essential 23.10 built in Colocalization Analyzer Advanced, using the integrated Costes method for background estimation^70,71^. As correlation metrics, the Object Pearson’s colocalization coefficient (range 0–1) and Object Spearman’s colocalization coefficient (range 0–1) were used because of their independence from background noise^71,72^.

### Modeling of cell shape and signal heterogeneity using advanced imaging analysis

To localize and quantify labeling signals on a subcellular scale, an in-depth machine learning (ML) analysis was applied using Imaris 10.1 software (Oxford Scientific Instruments, Abingdon, UK) at the CCB at *Medizinische Universität* Innsbruck. In particular, the surface function in Imaris, providing a trainable ML algorithm, was used. The ML algorithm was trained following the manufacturer’s instructions to detect and classify structures from the deconvolved confocal microscopy images to obtain object-to-object statistics (default setting). In the first step, cellular shape volumes were determined from AF signals by training using examples of regions with cellular AF signals (foreground) as well as from regions lacking cellular AF (background) under strong supervision^73^. Subsequent runs of the ML algorithm were performed until consistent detection and modeling of the cell shapes were achieved. In the second step, an additional algorithm was used analogously to model the heterogeneous A488-click labeling signals into so-called modeled entities (ME). For the quantitative analysis of the A488-click signal intensity, only ME above the intensity of 10 saturated voxels (2550 RFU ~ 0.00125 µm^3^) were considered (Fig. 4, Extended Data Figs. 2 and 3).

Owing to morphological differences between *M. aeruginosa* and *P. agardhii* (i.e., unicellular growth vs. growth in filaments), the training of the ML algorithm was performed separately for *M. aeruginosa* and *P. agardhii* using subsets of the confocal images under highest observed signal intensity, i.e., *M. aeruginosa* cells harvested from build-up experiments grown in the presence of Prop-Tyr for 168 h and *P. agardhii* filaments from signal decline experiments harvested at T0 after being grown in the presence of Prop-Lys for 48 h. Each of the algorithms was validated using previously unseen images under the same experimental conditions (same time-point and non-AA treatment) and cross-validated with new images under different experimental conditions. Model parameters were applied independently for *M. aeruginosa* and *P. agardhii*. Images from each non-AA treatment and time point were analyzed in batches. The quantitative results were subsequently extracted from Imaris using surface function (1) for the modeled cell shapes andsurface function (2) for ME.

The following Imaris statistical parameters were obtained for the ME: ME signal intensity (MEi), ME volume (MEv, in µm^3^), number of ME (MEn), ME surface area (µm^2^), minimum distance of ME to the modeled cell shape (mDtC, µm), overlapping volume of ME with the modeled cell shape (OV), center of mass position (ME center of mass X, Y, Z position), sphericity (Φ), and Euclidean distance to the image origin for the ME. The following parameters were obtained for modeled cell shapes: AF signal intensity, modeled shape volume (µm^3^), modeled cell shape center of mass position (X, Y, Z position) and sphericity (Φ).

### Calculating relative ME positions from imaging analysis parameters and classification into subcellular compartments

In a first step, the MEi and MEv percentages of the total cellular signal intensities were calculated (Extended Data Figs. 4 and 5) as well as the relationships between the cellular A488-click labeling intensity and the MEi were calculated (Extended Data Figs. 6 and 7). To obtain the relative ME positions in the cell, the distances between the centroids of individual ME and modeled cell shape surfaces (CtC) were calculated. For this purpose, the cell shape diameter for *M. aeruginosa* or cell shape height for *P. agardhii* were calculated from the cell shape volumes adjusted for sphericity (Φ). All ME with an OV ≥ 0.5 were considered to present a centroid located within the modeled cell shape for both *M. aeruginosa* (Extended Data Fig. 15) and *P. agardhii* (Extended Data Fig. 16). For those ME, the CtC distances were calculated by subtracting mDtC and the ME radius (MEr) from the modeled cell shape radius (Cr). In contrast, for ME with a lower OV (OV < 0.5), CtC distances were calculated under the assumption that the ME centroid was located outside of the modeled cell shape. Thus, the CtC distance was calculated as mDtC plus MEr plus Cr. Subsequently, the relative ME position was calculated by dividing the CtC distances of individual ME by the respective cell radius (*M. aeruginosa*) or height (*P. agardhii*), and either assigned to the cell membrane region (rel. pos. > 1) or to the cytoplasm (rel. pos. ≤ 1).

### Statistical analysis

Statistical analysis of all the quantitative data was performed by two-way repeated-measures ANOVA (1^st^ factor: time; 2^nd^ factor: non-AA treatment) for each cyanobacterium and time-lapse experiment separately. Statistical significance was inferred from p-values less than 0.05. For post hoc pairwise comparisons, p-values were adjusted using Bonferroni correction. All the statistical analyses were performed using R Studio 4.5.1. Regression lines (Figs. 2, 3, 5 and 6, and Extended Data Figs. 4, 5 and 8–14) were fitted from three technical replicates following the LOESS method.

To quantify the ME intracellular distances independent of the cell shape, a clustering analysis was performed from the ME 3D coordinates using the Mclust R package^74^ with the default settings. Individual ME were associated with a specific cluster according to their posterior probability within a Gaussian finite mixture model. Only ME assigned to abundant clusters, representing at least 1% of the total number of ME, were considered in the analysis. For each individual cluster distances between individual ME assigned to the cluster in one cell were obtained from the three-dimensional coordinates in the image (X, Y, Z), thus obtaining the Euclidean distances between the ME assigned to that cluster.

For *M. aeruginosa* a few dividing cells were manually selected to investigate if relative ME positions vary during cell division. For the statistical analysis, dividing cells were selected from the build-up (*n* = 92) and decline (*n* = 72) time-lapse experiments, and compared to an equal number of randomly selected unicellular *M. aeruginosa* cells, after excluding cells that may be in the initial stage of division. To reduce the effect of randomness in the statistical analysis, 1000 iterations of this selection were performed. A list of cell IDs undergoing division as well as those with unclear assignment is available in Bioimages Archive reference S-BSST2549.

## Supplementary Information

Below is the link to the electronic supplementary material.

Supplementary Material 1

Supplementary Material 2

## Data Availability

All the deconvolved original microscopy images used for this publication are available at the European Molecular Biology Laboratory (EMBI)’s BioImages Archive (https://www.ebi.ac.uk/bioimage-archive) under the accession number S-BIAD1325. The processed images resulting from the use of Imaris 10.1 software have been deposited under accession number S-BIAD1326. The immunofluorescence labeling images have been deposited under S-BIAD2489. The quantitative data extracted from Imaris analysis have been deposited in the EMBI’s BioStudies Archive under S-BSST2549 ( **M. aeruginosa** ) and S-BSST2550 ( **P. agardhii** ).
