# Publisher Correction: Subcellular visualization and quantification of cyanotoxin synthesis in cyanobacteria reveals distinct compartmentation

**DOI:** 10.1038/s41598-026-59560-1

**Published:** 2026-07-02

**Authors:** Rubén Morón-Asensio, Rainer Kurmayer

**Affiliations:** 1 Research Department for Limnology, University of Innsbruck, Mondseestrasse 9, 5310 Mondsee, Austria; 2 Universität Innsbruck, Innrain 52, 6020 Innsbruck, Austria

Correction to: *Scientific Reports* 10.1038/s41598-026-47303-1, published online 19 April 2026

The original version of this Article contained an error in Figs. 1 and 4 where rendering caused blurring of the images.

The original Figs. 1 and 4 and accompanying legends appear below.

**Fig. 1:**
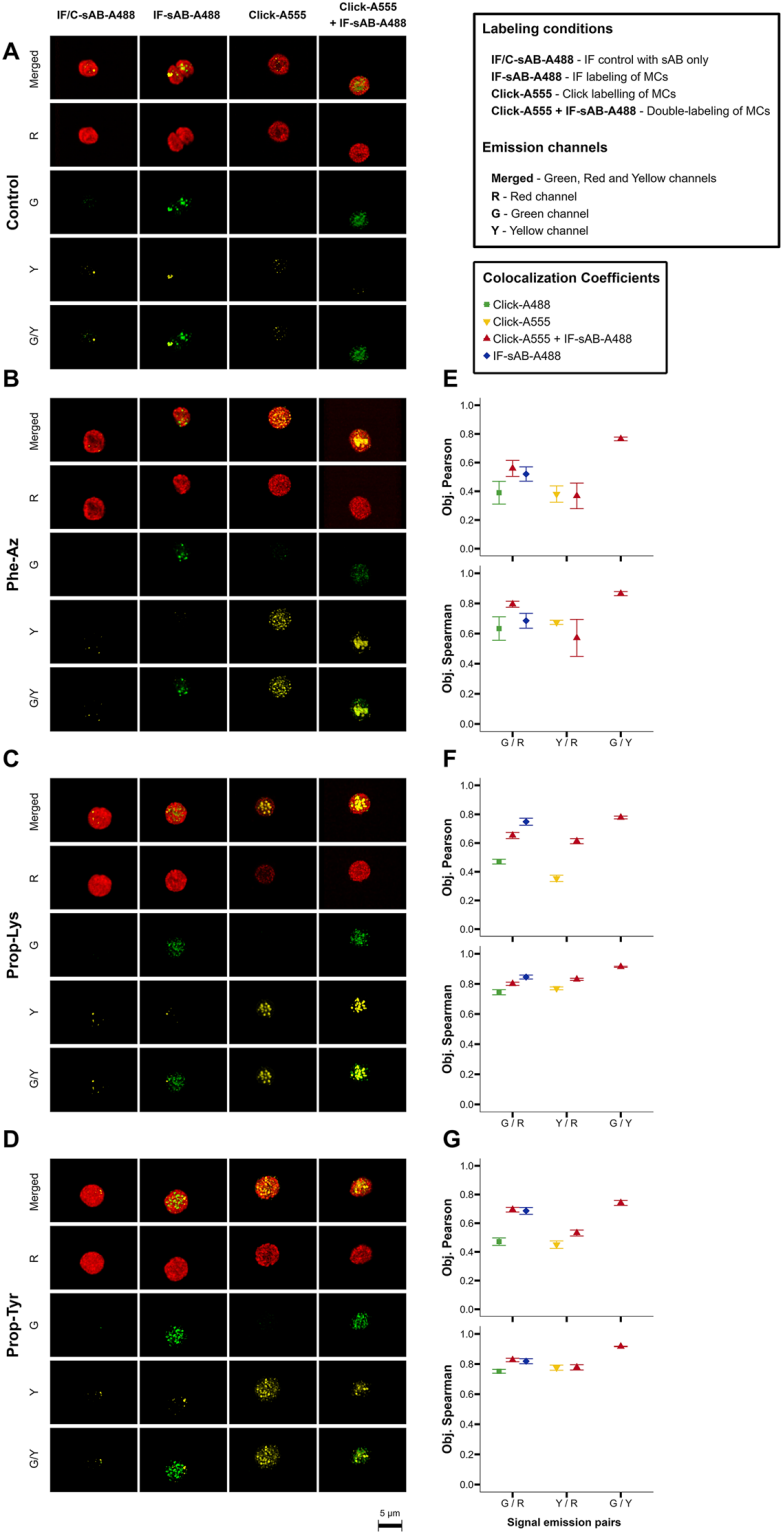
Combining immunofluorescence (IF) labeling of MCs with chemoselective visualization of MCs in cells of *M. aeruginosa* strain Hofbauer (**A**–**D**). For this purpose, a double-labeling protocol for chemoselective labeling of clickable MCs (yellow fluorophore, click-A555-azide/alkyne) and the subsequent immunolabeling of MCs (green fluorophore, IF-sAB-A488) was established. *M. aeruginosa* cells were grown in the presence of non-AA, either Phe-Az, or Prop-Lys, or Prop-Tyr, to enable a click-chemistry reaction between clickable MC and A555-azide/alkyne. Control cells were grown in the absence of non-AA but treated identically. For immunofluorescence a monoclonal primary antibody (binding to the Adda side chain of the MC molecule) coupled to sAB-488 was used. The specificity of the secondary antibody sAB-488 was tested in absence of primary antibody (IF/C). Natural autofluorescence (AF) is observed in red. (**E**–**G**) The mean ± SE colocalization coefficients (Object Pearson and Object Spearman) were calculated to quantify the spatial correlation between different emission pairs: green channel (G) vs. red channel (R), i.e., IF-sAB-A488 vs. AF; yellow channel (Y) vs. R, i.e., click-A555 vs. AF; G/Y, i.e., IF-sAB-A488 vs. click-A555. For quantitative comparison of signal intensity between G and Y for both MC and another protein (RbcL, Rubisco large subunit) see Extended Data Fig. 1.

**Fig. 4:**
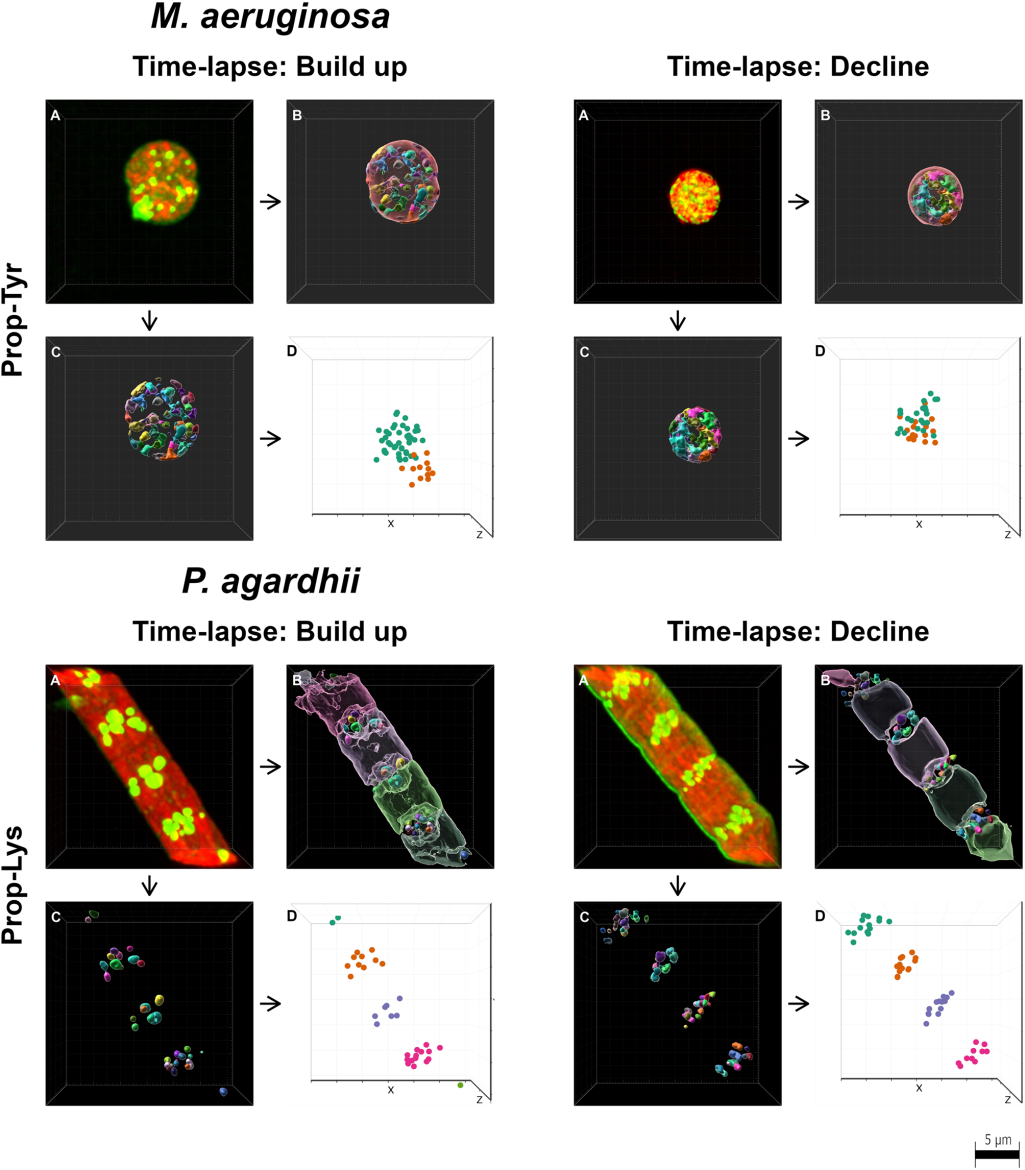
(**A**) Workflow for processing high-resolution 3D microscopy images of *M. aeruginosa* strain Hofbauer grown in the presence of Prop-Tyr to produce clickable MCs and *P. agardhii* strain No371/1 grown in the presence of Prop-Lys to produce clickable APs. (**B**) Autofluorescence of cells (red signal) was used to estimate the cell shape via Imaris surface function (1) (**C**) The A488-click signal (in green) was used to calculate modeled entities (ME) using Imaris surface function (2) (**D**) Clustering analysis of the ME (x, y, z) coordinates was performed to quantify the intracellular ME distribution. The analogous workflows for other non-AAs are shown in Extended Data Figs. 2 and 3.

The original Article has been corrected.

