# Supplementary material for: Subcellular visualization and quantification of cyanotoxin synthesis in cyanobacteria reveals distinct compartmentation
Source: Sci Rep. 2026 Apr 19;16:18148. doi: 10.1038/s41598-026-47303-1 (PMC13253841; doi:10.1038/s41598-026-47303-1)
Supplement: Supplementary file 2 — Supplementary Material 2 [file 41598_2026_47303_MOESM2_ESM.docx]

**Subcellular visualization and quantification of cyanotoxin synthesis in cyanobacteria reveals distinct compartmentation**

Rubén Morón Asensio^1,2^, Rainer Kurmayer^1,2*^

^1^ Research Department for Limnology, University of Innsbruck, Mondseestrasse 9, 5310 Mondsee, Austria; Ruben.Moron-Asensio@student.uibk.ac.at

^2^ Universität Innsbruck, Innrain 52, 6020 Innsbruck, Austria

^*^ Correspondence: [rainer.kurmayer@uibk.ac.at](mailto:rainer.kurmayer@uibk.ac.at) ; Tel.: 0043-512-507-50242

**Extended Data**


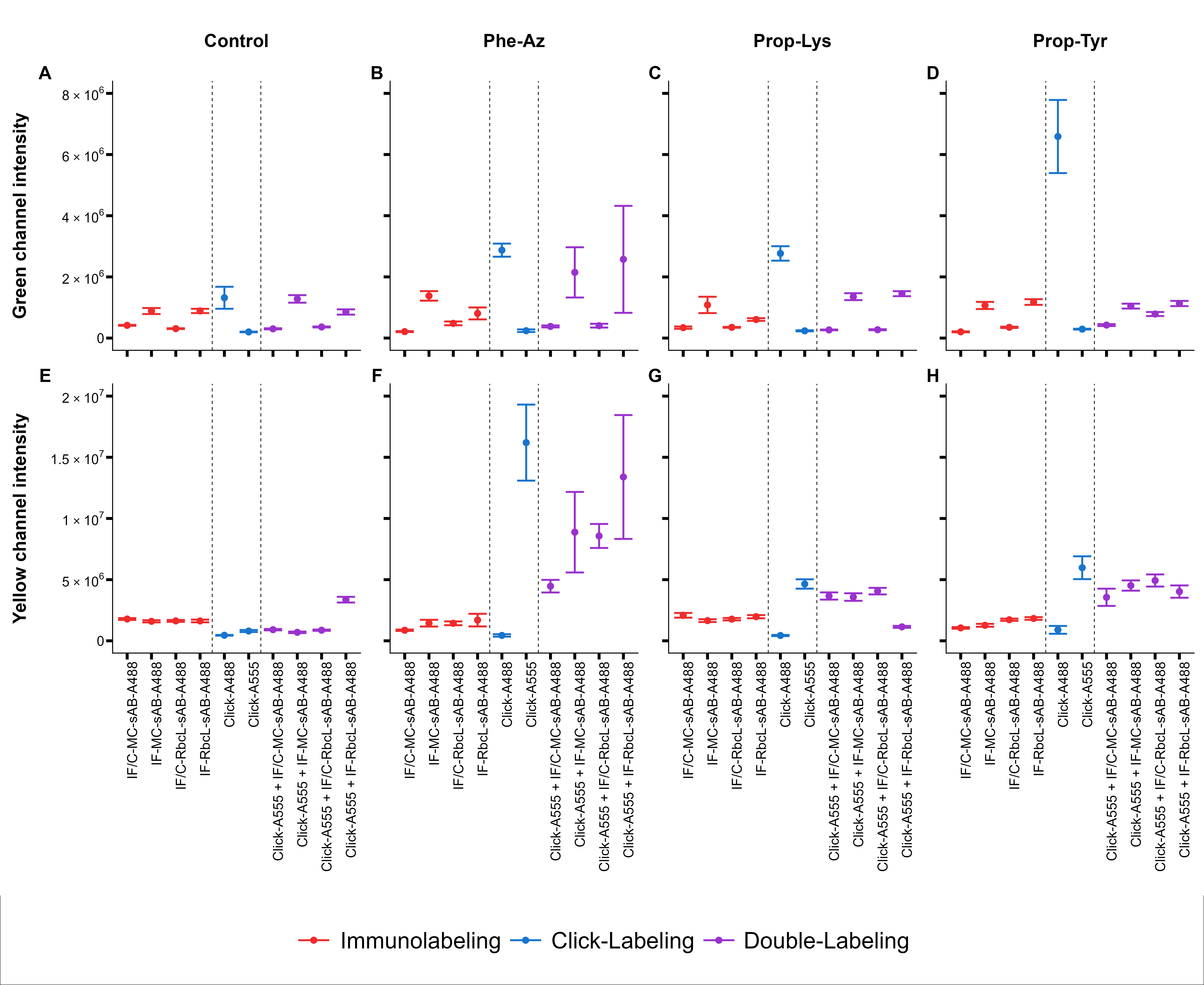


**Extended Data Fig. 1.** Comparison of signal intensity (mean ± SE) quantified in green channel (**A-D**) and yellow channel (**E-H**) for both MC and RbcL (Rubisco large subunit) in *M. aeruginosa* strain Hofbauer. Red symbols: Visualization of MC or RbcL by immunofluorescence labeling (IF) only. Blue symbols: Visualization of MC via chemoselective labeling only. Purple symbols: Visualization of MC or RbcL via double-labeling including firstly, chemoselective labeling of clickable MCs and secondly, immunolabeling of MCs. Dashed lines separate between the different labeling protocols applied. (**A-D**) Green channel (λ = 500 – 550 nm) labeling signal intensities in cells observed for the IF of MCs or RbcL using sAB-A488 or a click-chemistry reaction (A488-azide/alkyne) for the visualization of MCs. IF/C: In order to test unspecific binding only the secondary antibody was used. (**E-H**) Yellow channel (λ = 560 – 610 nm) labeling signal intensities (A555-click) for the visualization of MCs. Cells were grown in the presence of non-AAs (Phe-Az, Prop-Lys and Prop-Tyr) to enable chemoselective labeling either through A488-azide/alkyne or A555-azide/alkyne. Controls cells for chemoselective labeling were grown and processed under identical conditions but without non-AA substrate.


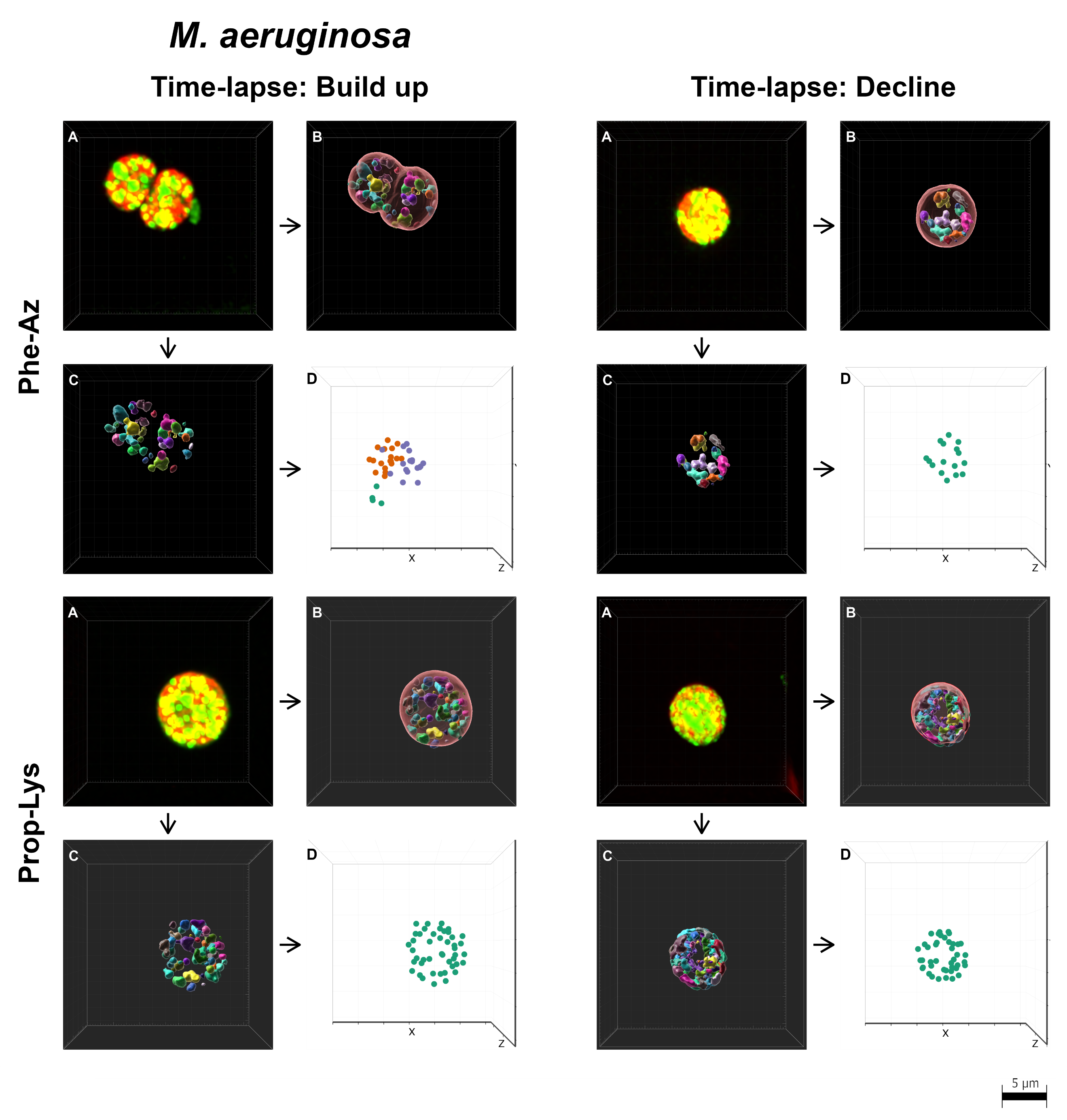


**Extended Data Fig. 2.** (**A**) Workflow for processing high-resolution 3D microscopy images of *M. aeruginosa* strain Hofbauer grown in the presence of Phe-Az and Prop-Lys to produce clickable MCs. (**B**) Autofluorescence of cells (red signal) was used to estimate the cell shape via Imaris surface function (1). (**C**) The A488 click signal (in green) was used to quantify modeled entities (ME) using Imaris surface function (2). (**D**) Clustering analysis of the ME (x, y, z) coordinates was performed to quantify the intracellular ME distribution.


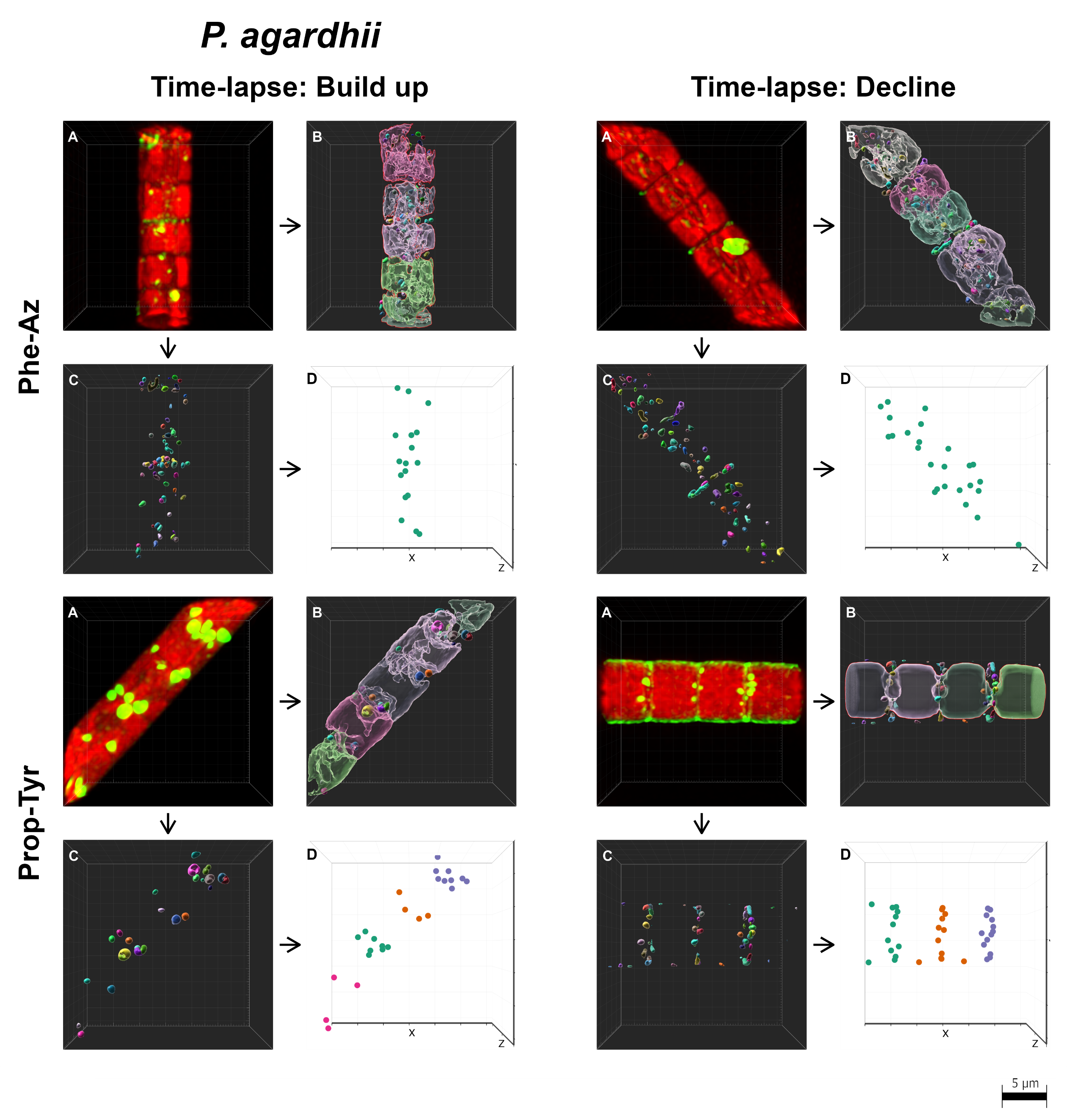


**Extended Data Fig. 3.** (**A**) Workflow for processing high-resolution 3D microscopy images of *P. agardhii* strain No371/1 grown in the presence of Phe-Az and Prop-Tyr to produce clickable APs. (**B**) Autofluorescence of cells (red signal) was used to estimate the cell shape via Imaris surface function (1). (**C**) The A488 click signal (in green) was used to calculate modeled entities (ME) using Imaris surface function (2). (**D**) Clustering analysis of the ME (x, y, z) coordinates was performed to quantify the intracellular ME distribution.


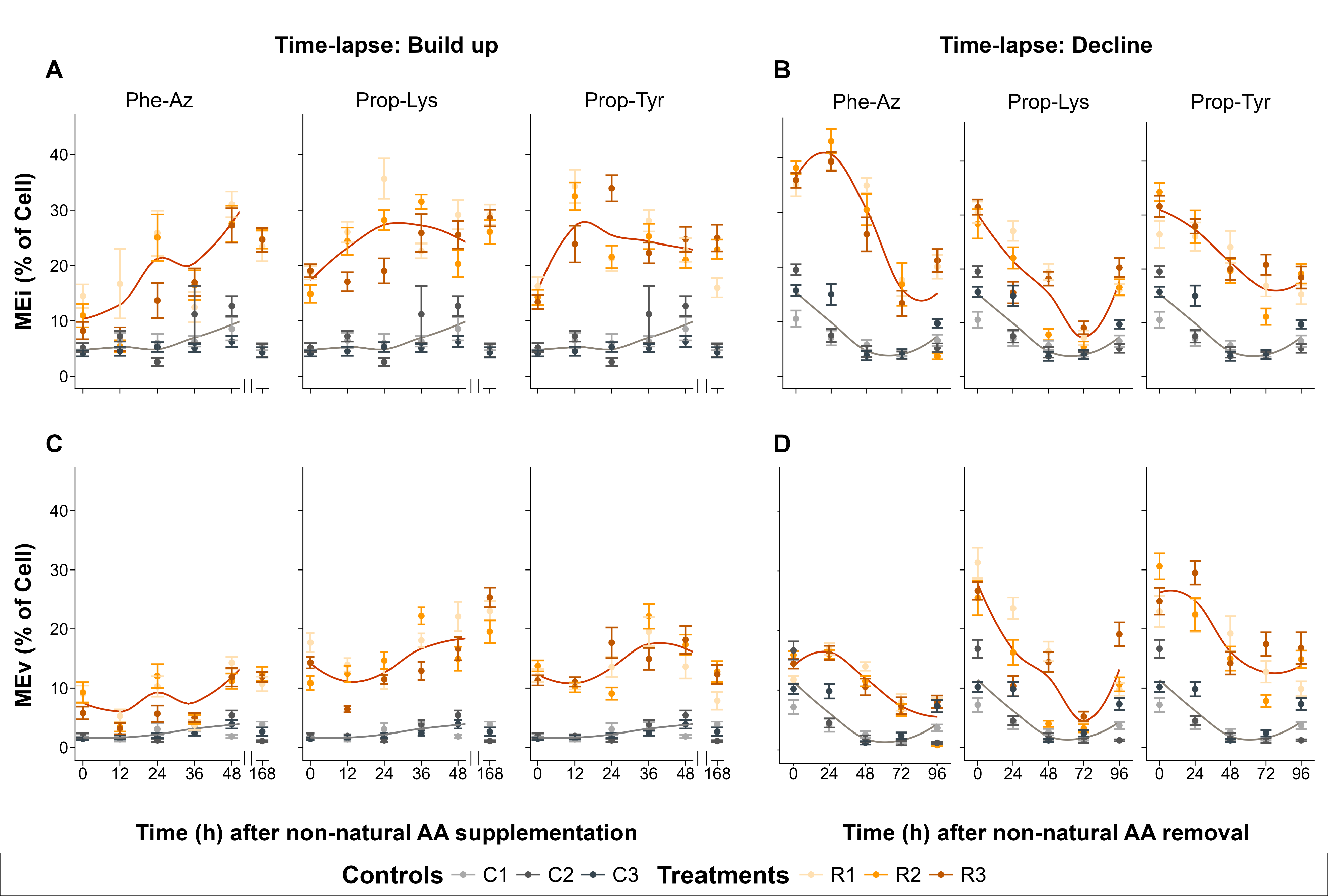


**Extended Data Fig. 4.** Quantification of signal intensity (mean ± SE) in modeled entities (ME) via advanced imaging analysis in *M. aeruginosa* strain Hofbauer during time-lapse experiments for build-up (**A**, **C**) or decline (**B**, **D**) using the pulsed feeding of non-AAs (Phe-Az, Prop-Lys, and Prop-Tyr) for clickable MCs production (non-AA treatments in orange and controls in black). (**A**, **B**) ME signal intensity (MEi) in percent of cellular signal intensity, (**C**, **D**) ME volume (MEv) in percent of cellular shape volume as quantified via AF.

**
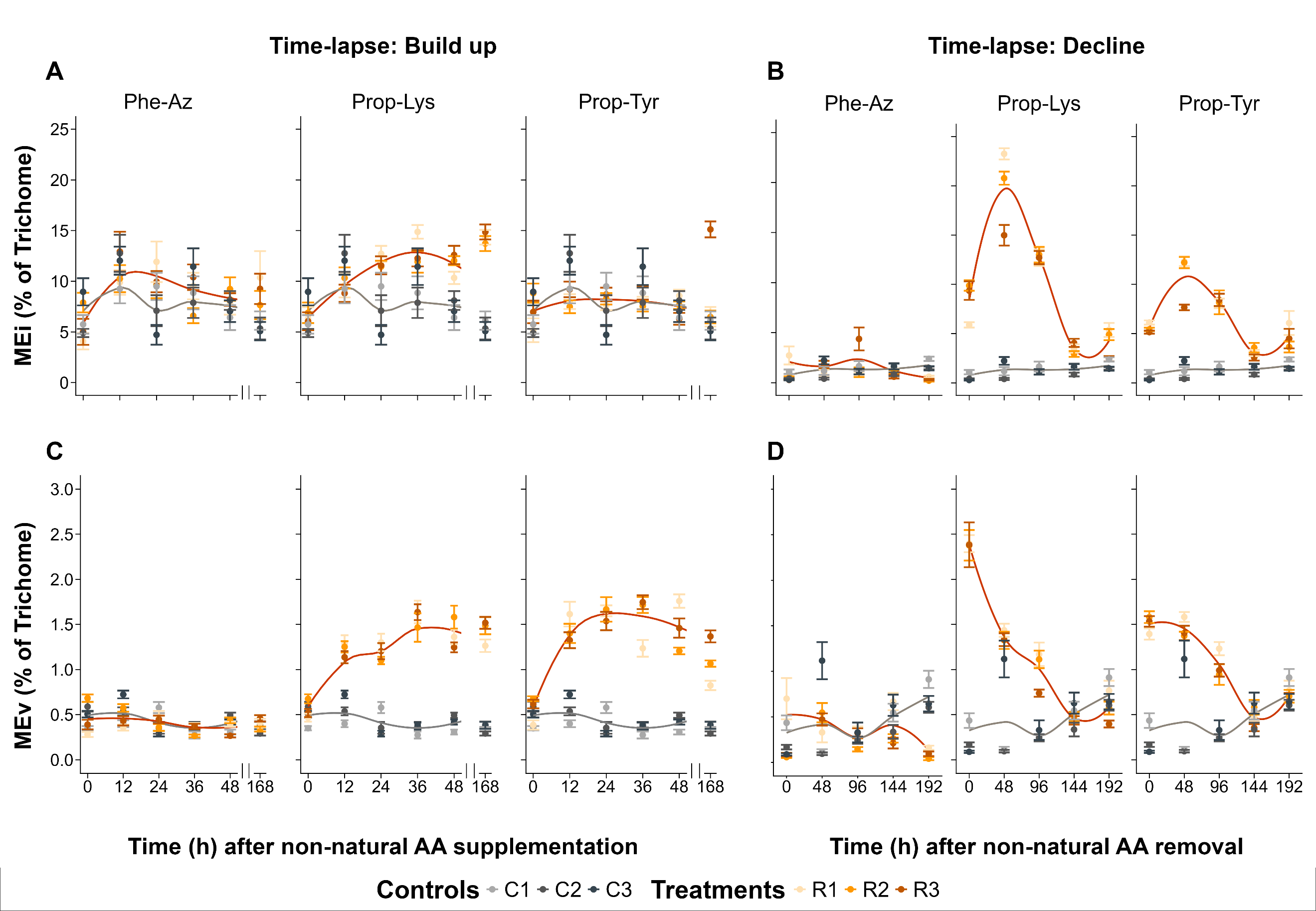
**

**Extended Data Fig. 5.** Quantification of signal intensity (mean ± SE) in modeled entities (ME) via advanced imaging analysis in *P. agardhii* strain No371/1 during time-lapse experiments for build-up (**A**, **C**) or decline (**B**, **D**) using the pulsed feeding of non-AAs (Phe-Az, Prop-Lys, and Prop-Tyr) for clickable APs production (non-AA treatments in orange and controls in black). (**A**, **B**) ME signal intensity (MEi) in percent of trichome signal intensity, (**C**, **D**) ME volume (MEv) in percent of trichome shape volume as quantified via AF.


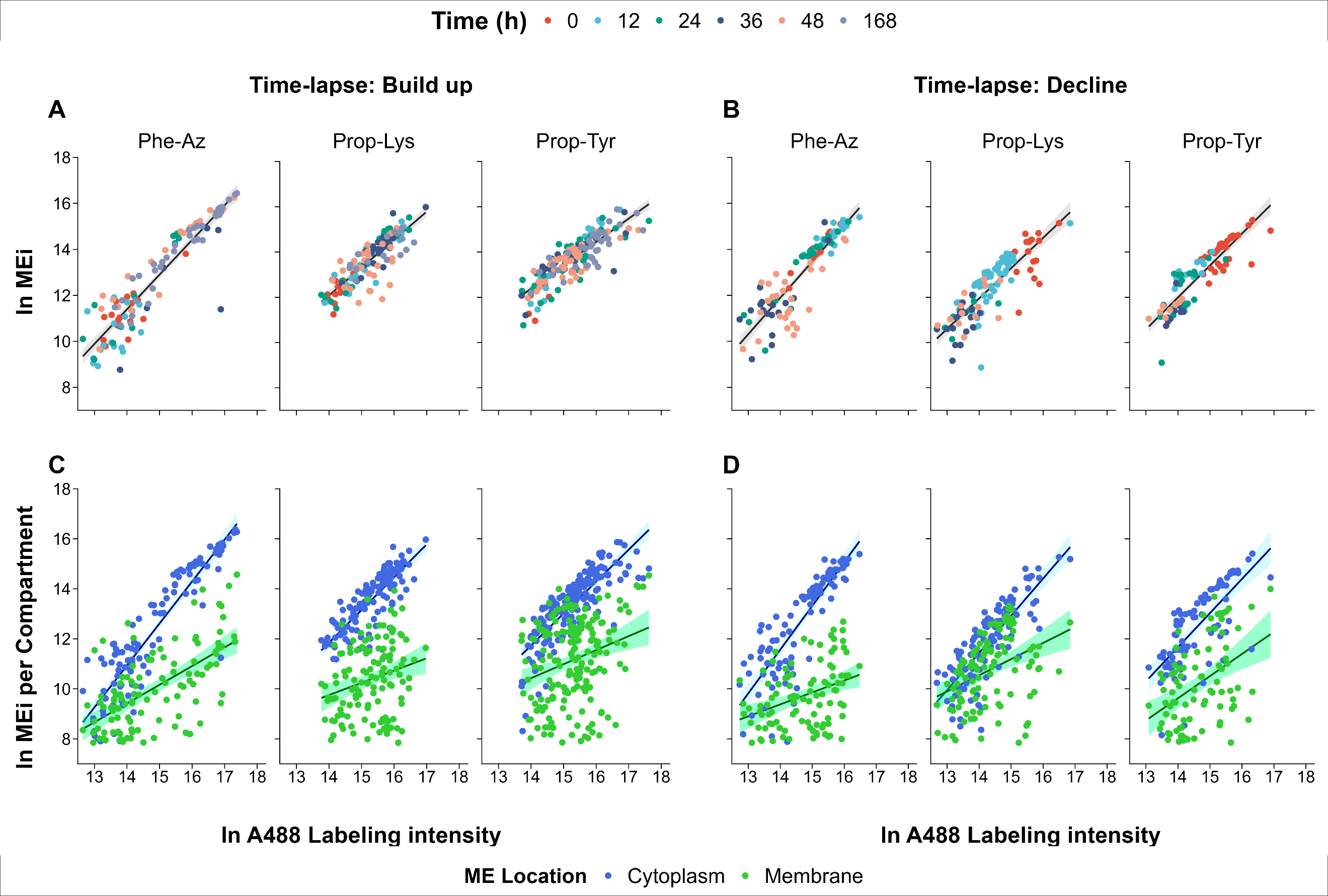


**Extended Data Fig. 6.** Relationship between cellular signal intensity from clickable MC and intensity quantified via so-called modeled entities (ME) resulting from advanced imaging analysis in *M. aeruginosa* strain Hofbauer during time-lapse experiments for build-up (**A**, **C**) or decline (**B**, **D**) using the pulsed feeding of non-AAs (Phe-Az, Prop-Lys, and Prop-Tyr) for clickable MCs production. (**A, B**) MEi vs. A488-click labeling intensity per cell (each color indicates a different time point); (**C, D**) MEi per compartment (cytoplasm, membrane region) vs. A488-click labeling intensity per cell.

Details of linear regression curves: (A) Phe-Az (y = 1.51x – 9.71, R^2^ = 0.85), Prop-Lys (y = 1.23x – 5.21, R^2^ = 0.71) and Prop-Tyr (y = 1.03x – 2.08, R^2^ = 0.71); (B) Phe-Az (y = 1.58x – 10.20, R^2^ = 0.80), Prop-Lys (y = 1.34x – 6.85, R^2^ = 0.70) and Prop-Tyr (y = 1.41x – 7.75, R^2^ = 0.78); (C) for the membrane region, Phe-Az (y = 0.75x – 1.06, R^2^ = 0.38), Prop-Lys (y = 0.49x + 2.88, R^2^ = 0.06) and Prop-Tyr (y = 0.56x + 2.59, R^2^ = 0.08); for the cytoplasm region, Phe-Az (y = 1.68x – 12.50, R^2^ = 0.83), Prop-Lys (y = 1.31x – 6.51, R^2^ = 0.64) and Prop-Tyr (y = 1.28x – 6.15, R^2^ = 0.63), (D) for the membrane region, Phe-Az (y = 0.48x + 2.68, R^2^ = 0.13), Prop-Lys (y = 0.65x + 1.51, R^2^ = 0.15) and Prop-Tyr (y = 0.89x – 2.77, R^2^ = 0.19); for the cytoplasm, Phe-Az (y = 1.75x – 12.90, R^2^ = 0.73), Prop-Lys (y = 1.49x – 9.37, R^2^ = 0.67) and Prop-Tyr (y = 1.37x – 7.45, R^2^ = 0.54), where y indicates ln of ME signal intensity and x indicates ln of A488-click signal intensity per cell.


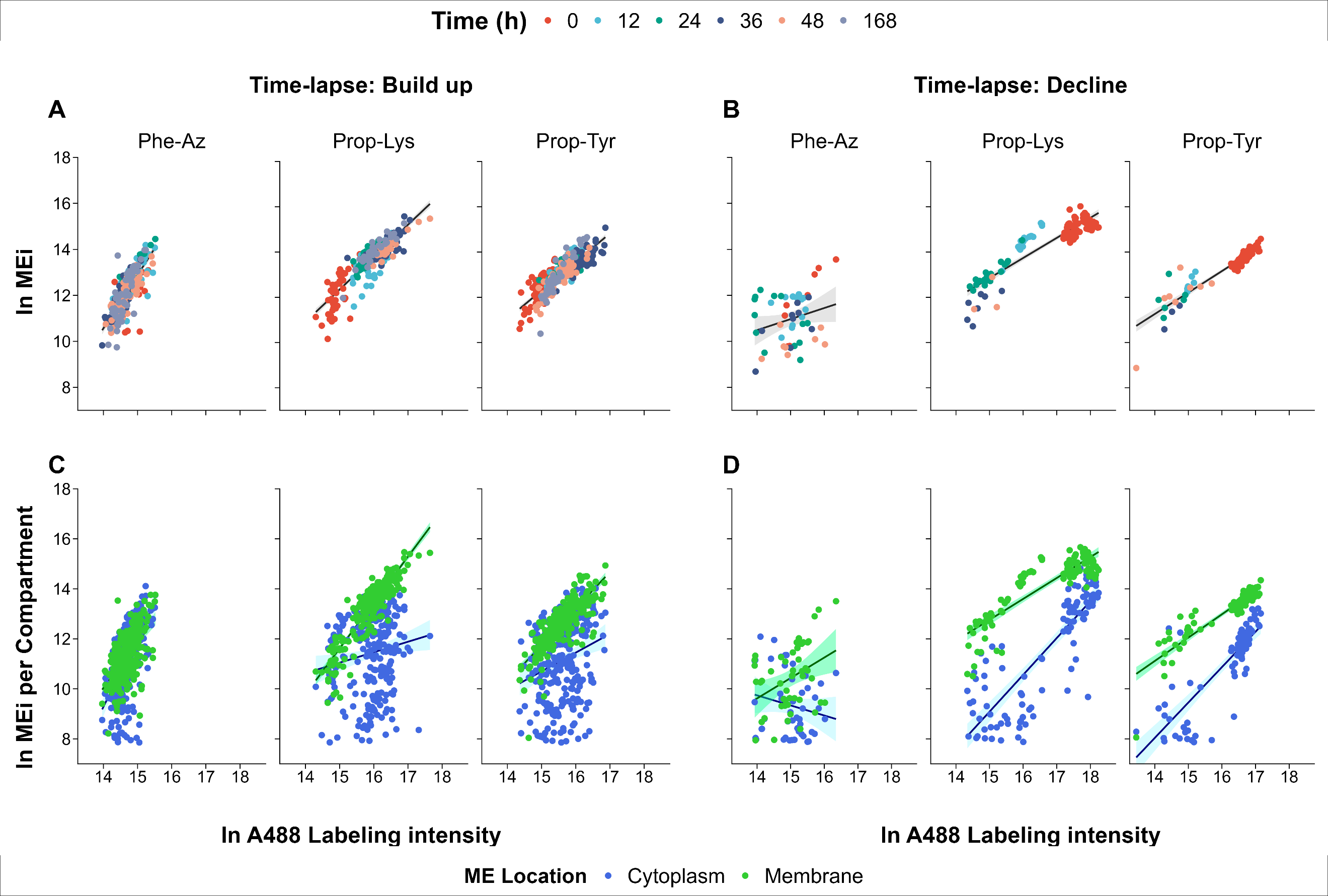


**Extended Data Fig. 7.** Relationship between cellular signal intensity from clickable AP and intensity via so-called modeled entities (ME) resulting from advanced imaging analysis in *P. agardhii* strain No371/1 during time-lapse experiments for build-up (**A**, **C**) or decline (**B**, **D**) using the pulsed feeding of non-AAs (Phe-Az, Prop-Lys, and Prop-Tyr) for clickable APs production. (**A, B**) MEi vs. A488-click labeling intensity per cell (each color indicates a different time point); (**C, D**) MEi per compartment (cytoplasm, membrane region) vs. A488-click labeling intensity per cell.

Details of linear regression curves: (A) Phe-Az (y = 2.32x – 21.90, R^2^ = 0.59), Prop-Lys (y = 1.43x – 9.14, R^2^ = 0.78) and Prop-Tyr (y = 1.26x – 6.61, R^2^ = 0.70); (B) Phe-Az (y = 0.75x -1.06, R^2^ = 0.38), Prop-Lys (y = 0.89x – 0.48, R^2^ = 0.82) and Prop-Tyr (y = 0.95x – 2.05, R^2^ = 0.85); (C) for the membrane region, Phe-Az (y = 1.82x – 15.50, R^2^ = 0.34), Prop-Lys (y = 1.84x – 15.90, R^2^ = 0.82) and Prop-Tyr (y = 1.46x – 10.20, R^2^ = 0.66); for the cytoplasm region, Phe-Az (y = 2.78x – 29.70, R^2^ = 0.34), Prop-Lys (y = 0.42x + 4.75, R^2^ = 0.02) and Prop-Tyr (y = 0.75x – 0.53, R^2^ = 0.06); (D) for the membrane region, Phe-Az (y = 0.82x – 1.82, R^2^ = 0.14), Prop-Lys (y = 0.84x + 0.21, R^2^ = 0.74) and Prop-Tyr (y = 0.93x – 1.97, R^2^ = 0.82); for the cytoplasm, Phe-Az (y = -0.40x + 15.40, R^2^ = 0.04), Prop-Lys (y = 1.47x – 13.00, R^2^ = 0.71) and Prop-Tyr (y = 1.42x – 11.80, R^2^ = 0.67), where y indicates ln of ME signal intensity and x indicates ln of A488-click signal intensity per cell.


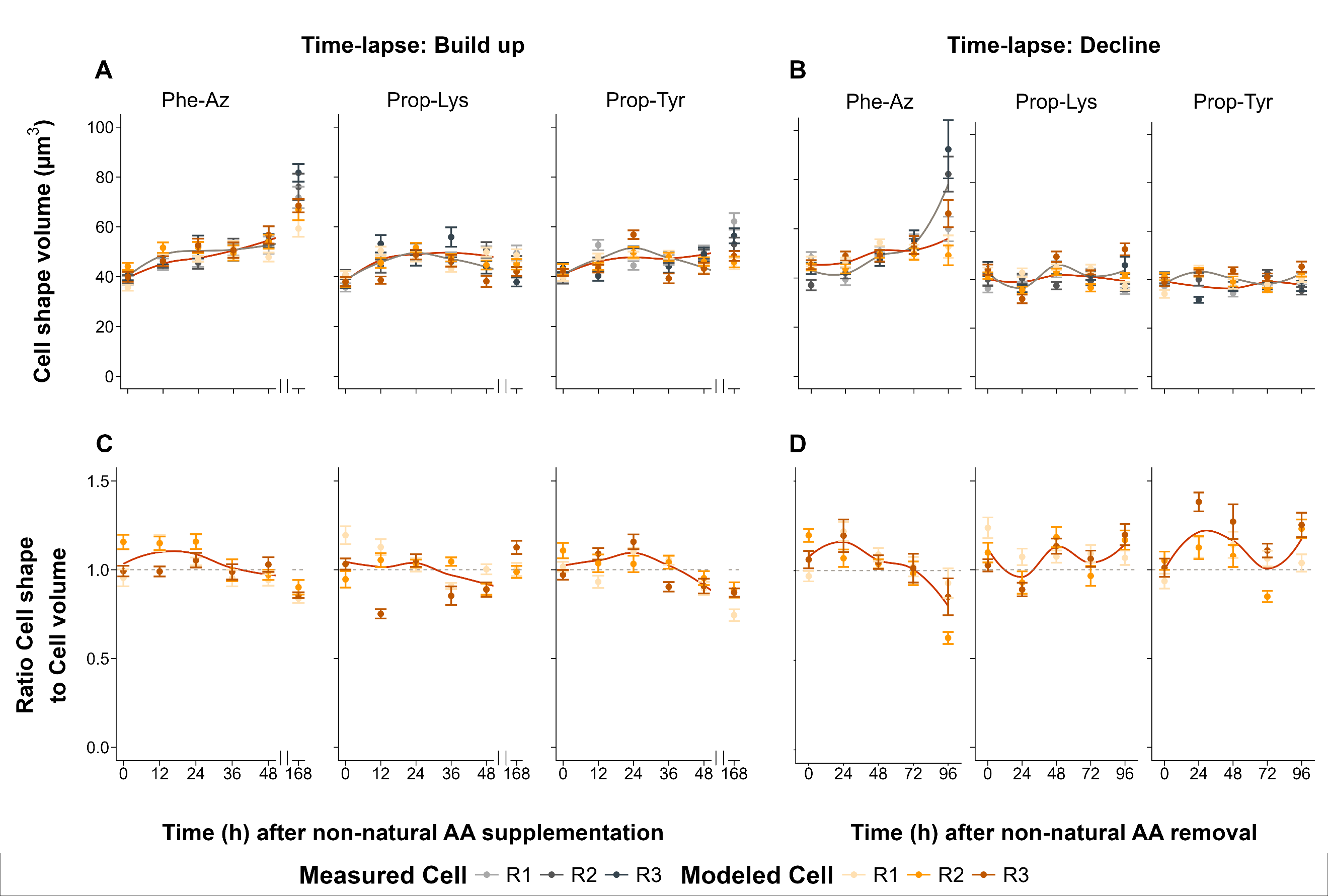


**Extended Data Fig. 8.** Visualization and quantification of *M. aeruginosa* strain Hofbauer cellular volumes (mean ± SE) during time-lapse experiments for build-up (**A**, **C**) or decline (**B**, **D**) as revealed by (**A**, **B**) modeled cell shape volume (orange) and measured cell shape volume via AF (black; calculated from the geometric shape of a sphere) and (**C**, **D**) ratio of modeled to observed cell shape volume during pulse feeding treatments with non-AAs (Phe-Az, Prop-Lys, and Prop-Tyr) for clickable MCs production.


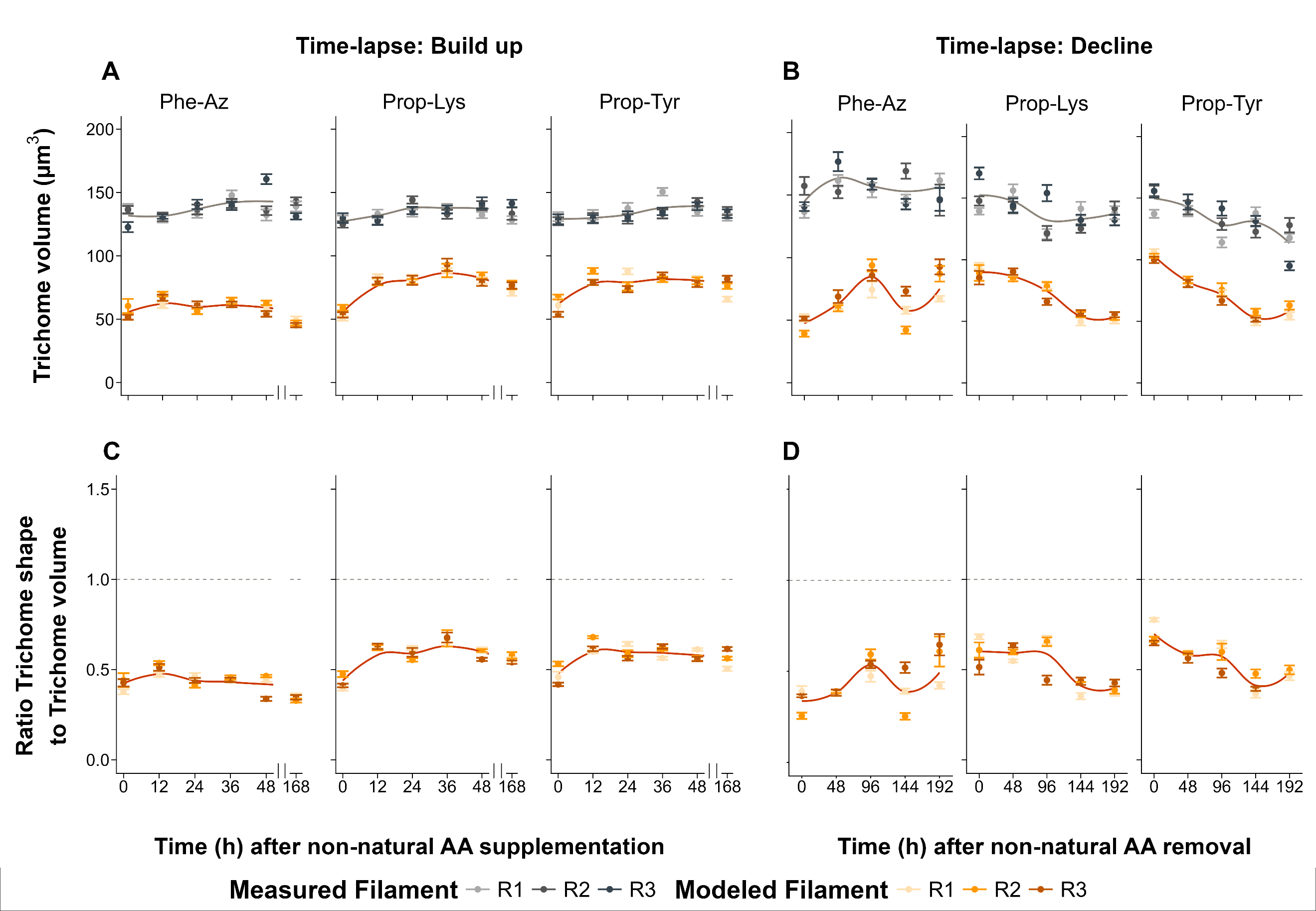


**Extended Data Fig. 9.** Visualization and quantification of *P. agardhii* strain No371/1 trichome volumes (mean ± SE) during time-lapse experiments for build-up (**A**, **C**) or decline (**B**, **D**) as revealed by (**A**, **B**) modeled volumes of individual trichomes (orange) and measured trichome shape volume via AF (black; calculated from the geometric shape of a cylinder) and (**C**, **D**) ratio of modeled to observed filament shape volume during pulse feeding treatments with non-AAs (Phe-Az, Prop-Lys, and Prop-Tyr) for clickable APs production.

**
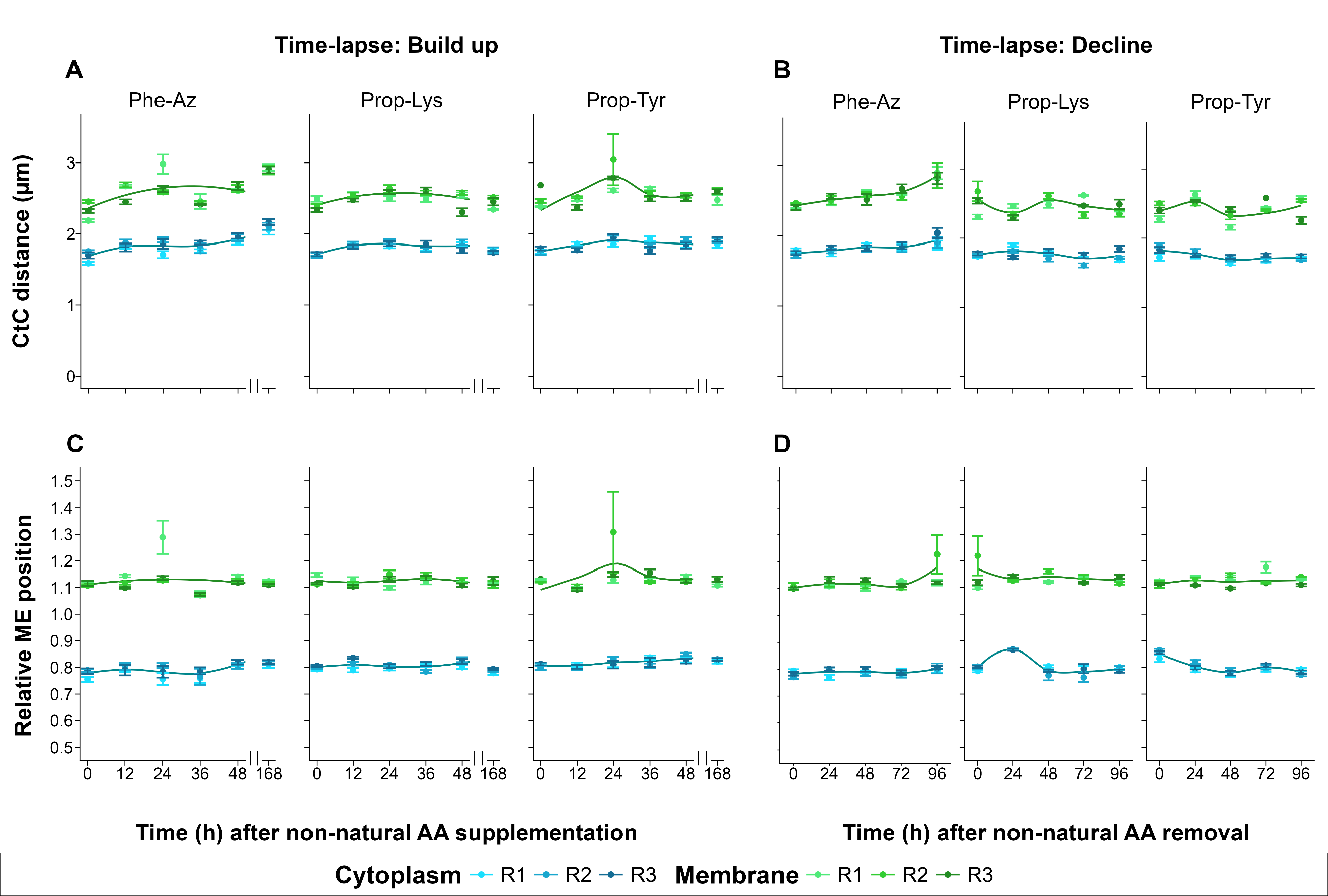
**

**Extended Data Fig. 10.** Compartmentation of cyanotoxin synthesis (mean ± SE) in *M. aeruginosa* strain Hofbauer during time-lapse experiments for build-up (**A**, **C**) or decline (**B**, **D**) as revealed by labeling of clickable MCs and advanced imaging analysis. (**A**, **B**) distance between ME and cell shape centroid (CtC) and (**C**, **D**) relative position of ME in two subcellular compartments (cytoplasm and membrane region) using the pulsed feeding of non-AAs (Phe-Az, Prop-Lys and Prop-Tyr) for clickable MCs production. Subcellular compartments were assigned according to the relative position of ME in the cell, i.e., in the cellular cytoplasm (blue; rel. pos. < 1) or in the membrane region (green; rel. pos. ≥ 1). Relative positions were calculated by dividing the CtC by the measured cell radius (see also Extended Data Fig. 15).


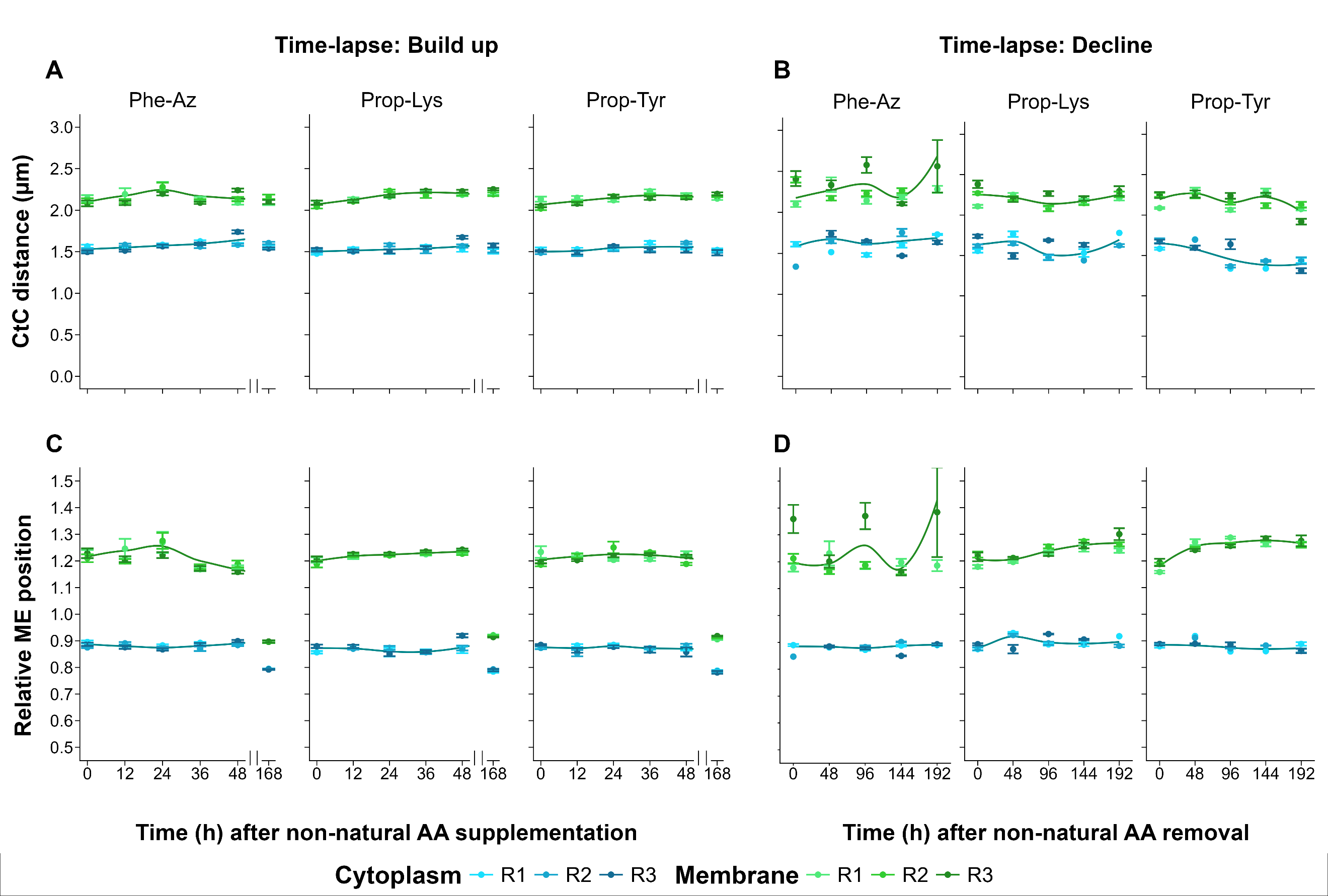


**Extended Data Fig. 11.** Compartmentation of cyanotoxin synthesis (mean ± SE) in *P. agardhii* strain No371/1 during time-lapse experiments for build-up (**A**, **C**) or decline (**B**, **D**) as revealed by labeling of clickable APs and advanced imaging analysis. (**A**, **B**) distance between ME and cell shape centroid (CtC) and (**C**, **D**) relative position of ME in two subcellular compartments (cytoplasm and membrane region) using the pulsed feeding of non-AAs (Phe-Az, Prop-Lys and Prop-Tyr) for clickable APs production. Subcellular compartments were assigned according to the relative position of ME in the cell, i.e., in the cellular cytoplasm (blue; rel. pos. < 1) or in the membrane region (green; rel. pos. ≥ 1). Relative positions were calculated by dividing the CtC by the measured cell radius (see also Extended Data Fig. 16).


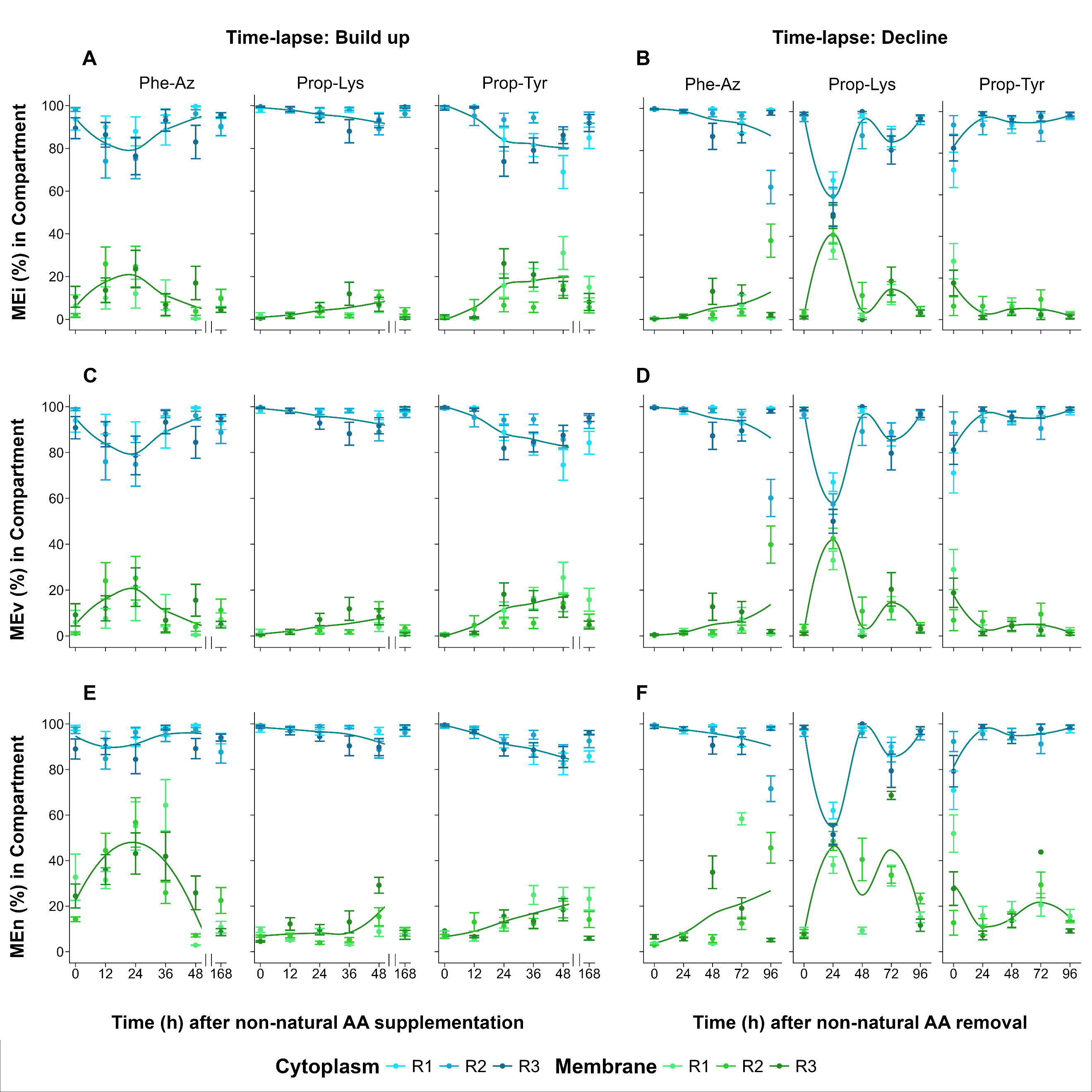


**Extended Data Fig. 12.** Compartmentation of cyanotoxin synthesis (mean ± SE) in *M. aeruginosa* strain Hofbauer during time-lapse experiments for build-up (**A**, **C**, **E**) or decline (**B**, **D**, **F**) as revealed by labeling of clickable MCs and advanced imaging analysis. (**A**, **B**) ME signal intensity (MEi) in percent of total MEi, (**C**, **D**) ME volume (MEv) in percent of total MEv and (**E**, **F**) ME numbers (MEn) in percent of total MEn in two subcellular compartments (cytoplasm vs. membrane region) using the pulsed feeding of non-AAs (Phe-Az, Prop-Lys and Prop-Tyr) for clickable MCs production. Subcellular compartments were assigned according to the relative position of ME in the cell, i.e., in the cellular cytoplasm (blue; rel. pos. < 1) or in the membrane region (green; rel. pos. ≥ 1).


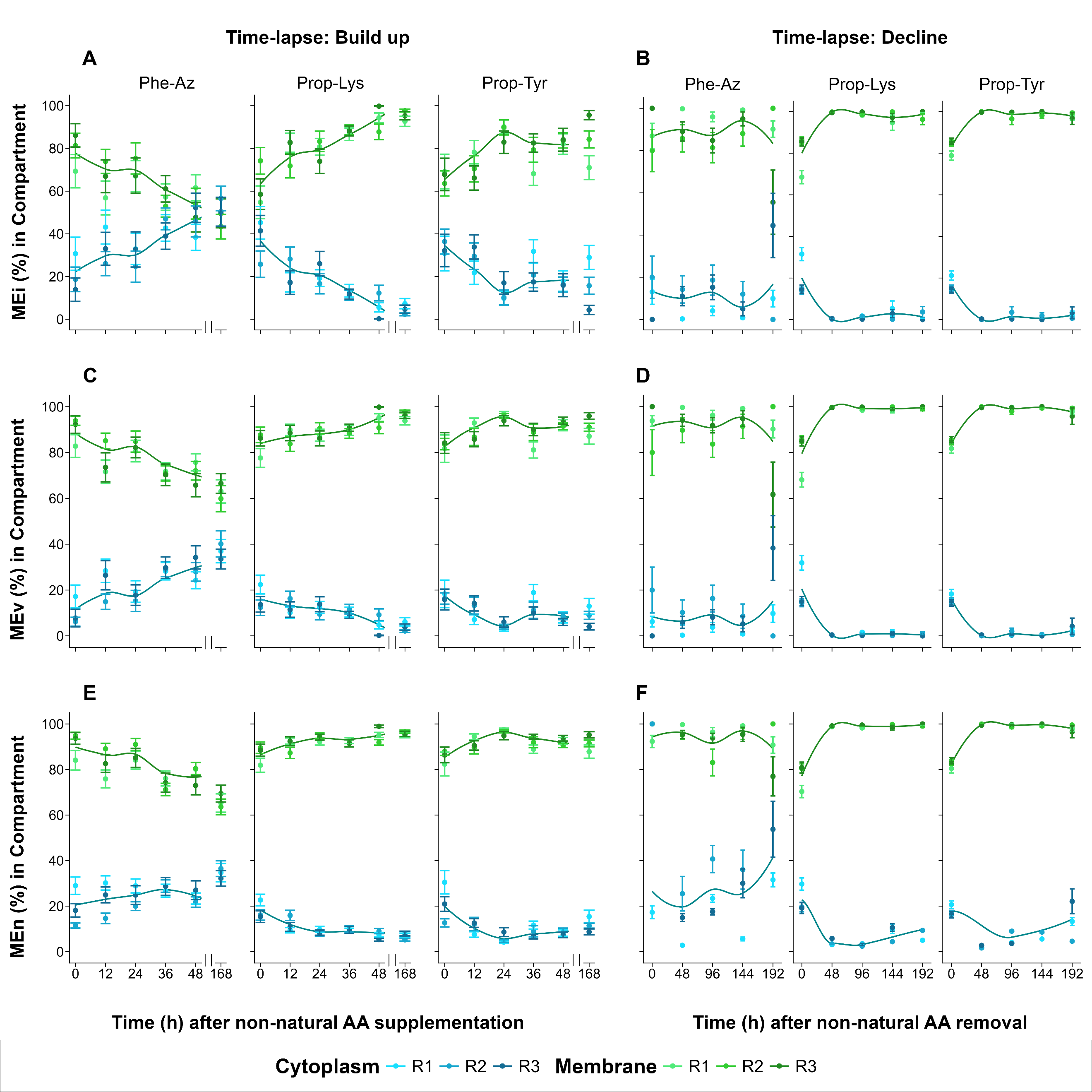


**Extended Data Fig. 13.** Compartmentation of cyanotoxin synthesis (mean ± SE) in *P. agardhii* strain No371/1 during time-lapse experiments for build-up (**A**, **C**, **E**) or decline (**B**, **D**, **F**) as revealed by labeling of clickable APs and advanced imaging analysis. (**A**, **B**) ME signal intensity (MEi) in percent of total MEi, (**C**, **D**) ME volume (MEv) in percent of MEv and (**E**, **F**) ME numbers (MEn) in percent of total MEn in two subcellular compartments (cytoplasm vs. membrane region) using the pulsed feeding of non-AAs (Phe-Az, Prop-Lys and Prop-Tyr) for clickable APs production. Subcellular compartments were assigned according to the relative position of ME in the cell, i.e., in the cellular cytoplasm (blue; rel. pos. < 1) or in the membrane region (green; rel. pos. ≥ 1).


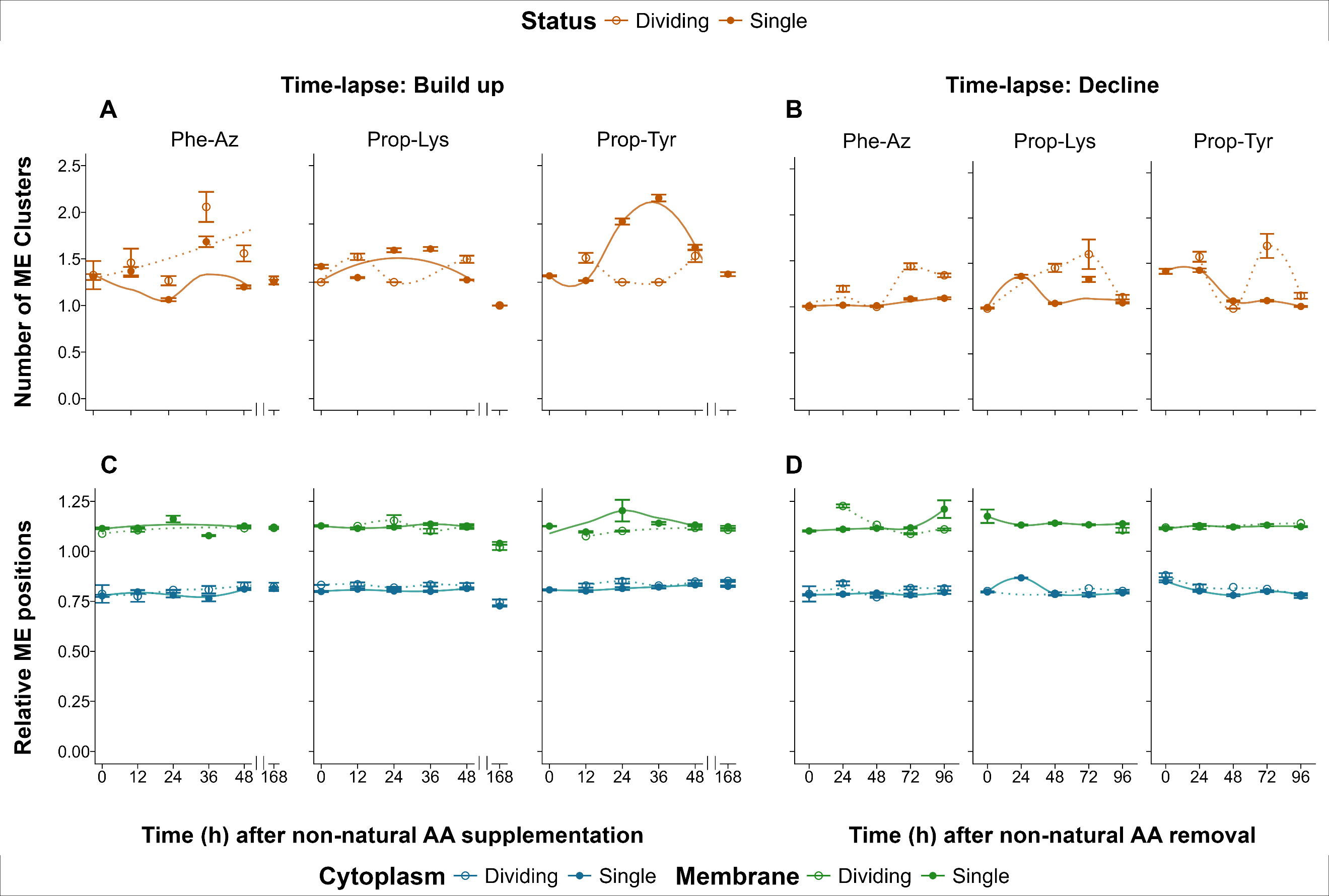


**Extended Data Fig. 14.** Compartmentation of cyanotoxin synthesis (mean ± SE) in dividing (open circles) vs. single (solid circles) of *M. aeruginosa* strain Hofbauer cells during time-lapse experiments for build-up (**A**, **C**) or decline (**B**, **D**) as revealed by labeling of clickable MCs and advanced imaging analysis. (**A**, **B**) number of clusters per cell (orange) and (**C**, **D**) relative ME positions in the cell for two subcellular compartments (cytoplasm vs. membrane region) using the pulsed feeding of non-AAs (Phe-Az, Prop-Lys and Prop-Tyr) for clickable MCs production. Regression lines were fitted distinctively for dividing cells (dotted lines) or single cells (solid lines). Subcellular compartments were assigned according to the relative position of ME in the cell, i.e., for the cellular cytoplasm (blue; rel. pos. < 1) or for the membrane region (green; rel. pos. ≥ 1).

**
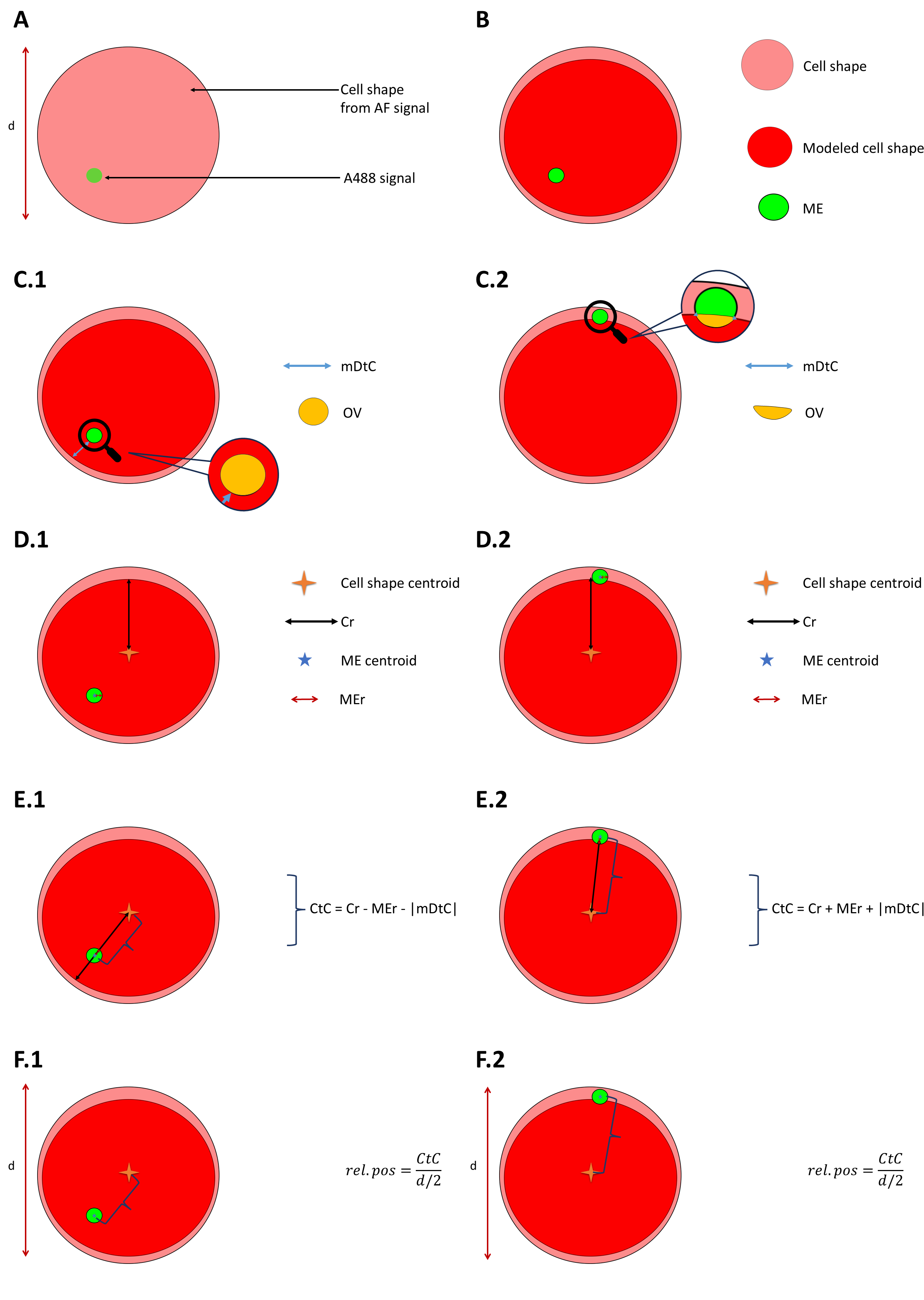
**

**Extended Data Fig. 15.** Scheme of ME parameter estimation for *M. aeruginosa*. **(A)** Original image obtained for *M. aeruginosa* cells via the AF and A488-click signals. The cell diameter was determined by measuring the cells from the AF in the original images. **(B)** Modeled cell shapes and modeled entities (ME) were estimated using Imaris 10.1 surface function, and cell shape and cell shape volumes were estimated from the AF. (**C)** Two ME parameters were obtained: the minimum distance (mDtC) between the cell shape surface and ME surfaces and their overlapping volume (OV). Two categories for ME positions were distinguished: **C.1** with high OV (≥ 0.5), indicating ME located within the cell shape, or **C.2** with a low OV (< 0.5), indicating ME located at the periphery of the cell shape. (**D.1, D.2**) After adjusting for sphericity (Φ), the modeled cell shape radius (Cr) and ME radius (MEr) were used to calculate the cell shape and ME centroid. Cr was calculated using $Cr=\left( 6*\mathrm{Vol}_{\mathrm{cell}}/\pi*\Phi\right)^{\frac{1}{3}}$, and MEr was calculated using $MEr=\left( 6*MEv/\pi*\Phi\right)^{\frac{1}{3}}$. (**E**) Distances between the modeled cell shape centroid and ME centroid (CtC) were calculated from the parameters mDtC, OV, Cr and MEr. **(E.1)** For ME with an OV ≥ 0.5 (indicating ME located within the cell shape), CtC was calculated using $CtC=Cr-MEr-|mDtC|$. (**E.2**) For ME with a low OV (indicating ME located outside the cell shape), CtC was calculated using $CtC=Cr+MEr+|mDtC|$. **(F)** The relative positions of ME with a high OV (**F.1**) or low OV (**F.2**) were calculated as the CtC divided by the cell shape radius.
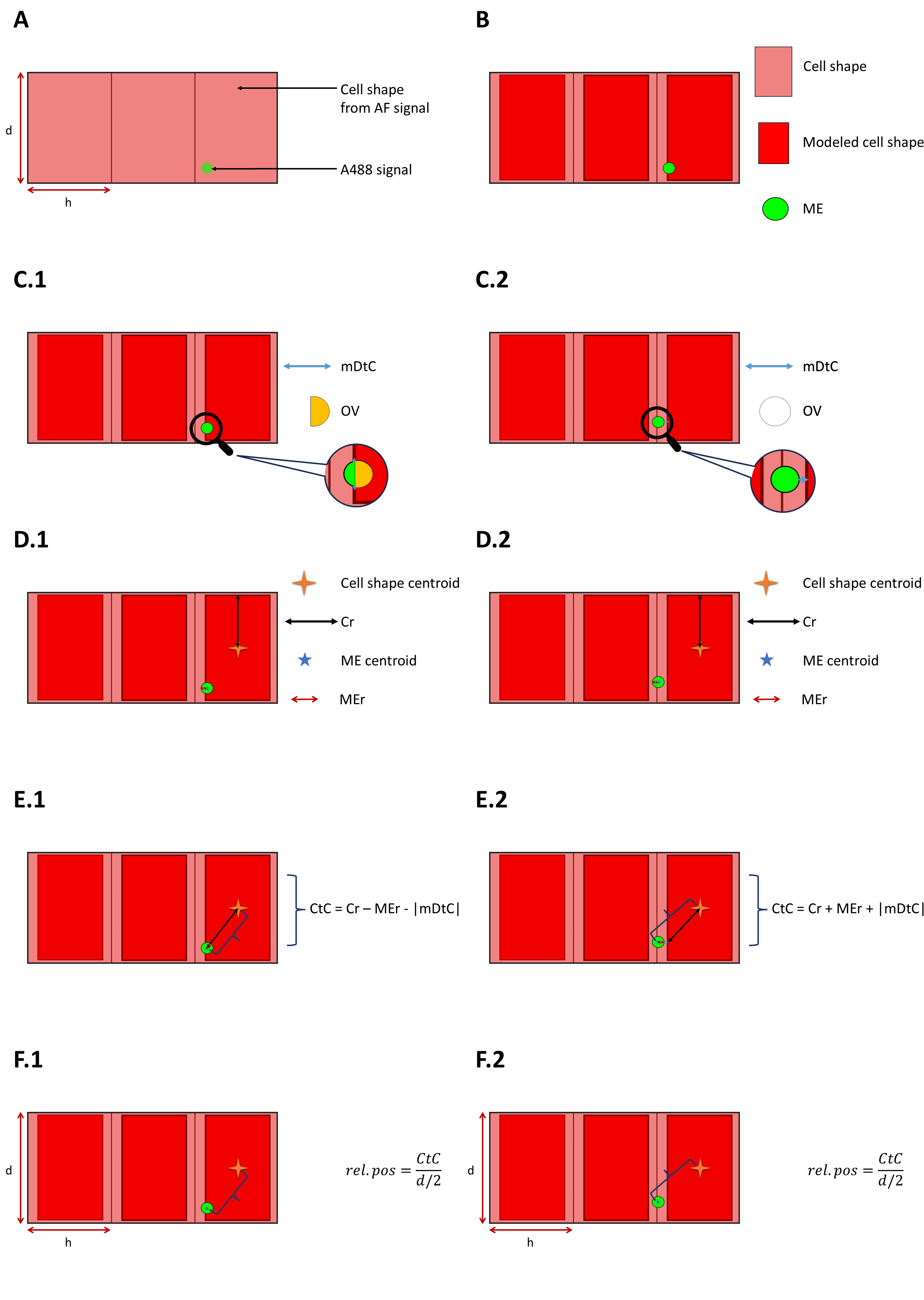


**Extended Data Fig. 16.** Scheme of ME parameter estimation for *P. agardhii*. **(A)** Original image obtained for *P. agardhii* filaments via AF and A488-click signals. The cell diameter was determined by measuring the cells from the AF in the original images. **(B)** Modeled cell shapes and modeled entities (ME) were estimated using Imaris 10.1 surface function and cell shape and cell shape volumes were estimated from the AF. The height of individual cells in the trichome was estimated from the modeled cell shape volume: $h= {V_{cell}}/\left[ {\pi*{(d}/{2)}}^{2} \right].$ **(C)** Two ME parameters were obtained: the minimum distance (mDtC) between the cell shape surface and ME surfaces and their overlapping volume (OV). Two categories for ME positions were distinguished: **C.1** with a high OV (≥ 0.5), indicating the ME located within the cell shape, or **C.2** with low OV (< 0.5), indicating ME located at the periphery of the cell shape. (**D.1, D.2**) After adjusting for sphericity (Φ), the modeled cell shape radius (Cr) and ME radius (MEr) were used to calculate the cell shape and ME centroid. Cr was calculated using $Cr=\left( 6*\mathrm{Vol}_{\mathrm{cell}}/\pi*\Phi\right)^{\frac{1}{3}}$, and MEr was calculated using $MEr=\left( 6*MEv/\pi*\Phi\right)^{\frac{1}{3}}$. (**E**) Distances between the modeled cell shape centroid and ME centroid (CtC) were calculated from the parameters mDtC, OV, Cr and MEr. **(E.1)** For ME with an OV ≥ 0.5 (indicating ME located within the cell shape), CtC was calculated using $CtC=Cr-MEr-|mDtC|$. (**E.2**) For ME with low OV (indicating ME located outside the cell shape), CtC was calculated using $CtC=Cr+MEr+|mDtC|$. **(F)** The relative positions of ME with a high OV (**F.1**) or low OV (**F.2**) were calculated as the CtC divided by the cell shape radius.
